# Youthfulness of marrow Adipoq^+^ cells maintained by Cbfβ facilitates stem cell-based bone repair

**DOI:** 10.1038/s41413-026-00568-8

**Published:** 2026-08-10

**Authors:** Tiannan Huang, Shali Wu, Wei Qian, Ruiying Chen, Erman Chen, Meizhu Wang, Cui Zhang, Chenhe Zhou, Luyang Yu, Mengjie Wu, Yi-Ping Li, Mengrui Wu

**Affiliations:** 1 Department of Cell and Developmental Biology, College of Life Sciences, Zhejiang University, Hangzhou, Zhejiang, China; 2 Department of Oral and Maxillofacial Implantology, Shanghai Ninth People’s Hospital, Shanghai Jiao Tong University School of Medicine; College of Stomatology, Shanghai Jiao Tong University; National Center for Stomatology; National Clinical Research Center for Oral Diseases; Shanghai Key Laboratory of Stomatology; Shanghai Research Institute of Stomatology, Shanghai, China; 3 Department of Orthopedics Surgery, The Second Affiliated Hospital, Zhejiang University School of Medicine, Hangzhou, Zhejiang, China; 4 Zhejiang University, Eye Center of Second Affiliated Hospital, School of Medicine, Zhejiang Provincial Key Laboratory of Ophthalmology; Zhejiang Provincial Clinical Research Center for Eye Diseases; Zhejiang Provincial Engineering Institute on Eye Diseases, Hangzhou, Zhejiang, China; 5 Institute of Genetics and Regenerative Biology, College of Life Sciences, Zhejiang University, Hangzhou, Zhejiang, China; 6 Stomatology Hospital, School of Stomatology, Zhejiang University School of Medicine, Zhejiang Provincial Clinical Research Center for Oral Diseases, Hangzhou, Zhejiang, China; 7 Key Laboratory of Oral Biomedical Research of Zhejiang Province, Cancer Center of Zhejiang University, Engineering Research Center of Oral Biomaterials and Devices of Zhejiang Province, Hangzhou, Zhejiang, China; 8 Division in Cellular and Molecular Medicine, Department of Pathology and Laboratory Medicine, Tulane University School of Medicine, Tulane University, New Orleans, LA, USA

**Keywords:** Bone, Metabolic disorders

## Abstract

Exhaustion of skeletal stem and progenitor cells (SSPCs) drives age-related delays in fracture repair, yet the upstream regulators of SSPC maintenance are unclear. We identify that core-binding factor β (Cbfβ) in bone marrow Adipoq^+^ cells (BMACs) is essential for maintaining SSPC number and function. Cbfβ deletion in BMACs (CKO) leads to SSPC depletion, including periosteal populations, and impairs bicortical fracture healing in mice. Multi-omics (RNA-seq, CUT&Tag-seq, and ATAC-seq) reveal that Cbfβ preserves chromatin accessibility at DNA repair loci, maintaining genomic stability, preventing BMAC senescence, and mitigating the senescence-associated secretory phenotype (SASP). Senolytic therapy alleviates BMAC senescence, restores SSPC populations, and improves bone repair in CKO mice. In both humans and mice, Cbfβ expression declines with aging, accompanied by increased BMAC senescence. AAV-mediated Cbfβ overexpression restores aging-related bone repair and SSPC decline. These findings reveal a novel mechanism in which Cbfβ in BMACs regulates SSPC maintenance via a senescence/SASP axis, offering a potential therapeutic strategy for age-related bone repair deficits.

**Figure:**
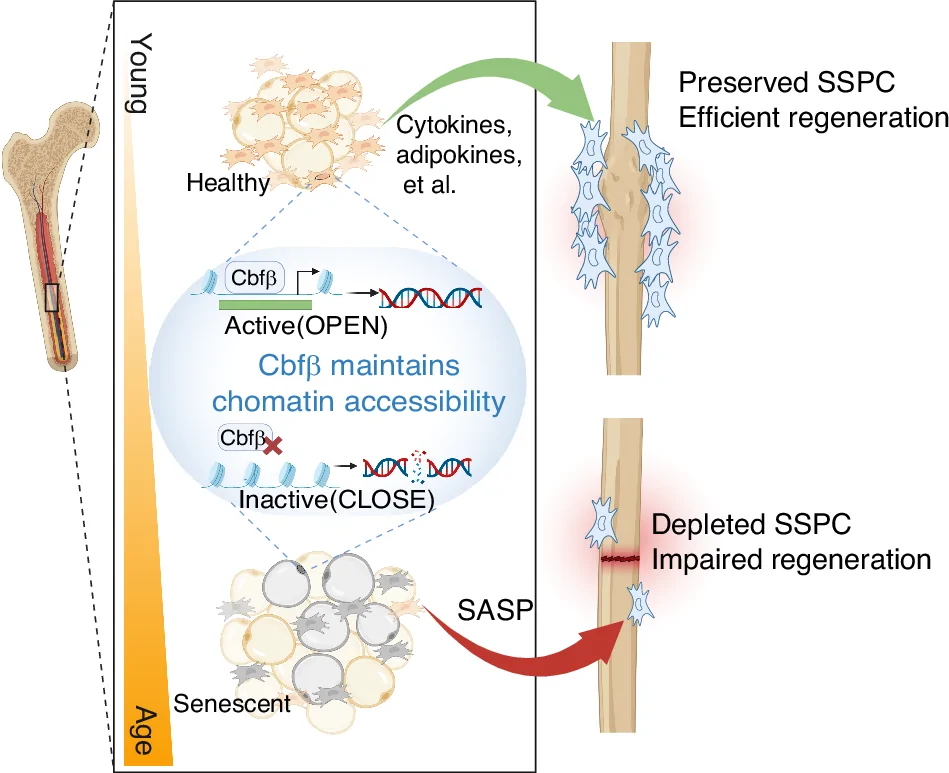

## Introduction

Delayed fracture healing and an increased incidence of nonunion are major contributors to the high disability and mortality rates associated with fractures in the elderly.^1,2^ Fracture repair is a complex process that requires the recruitment of skeletal stem and progenitor cells (SSPCs), which sequentially differentiate into chondrocytes and osteoblasts to form a cartilaginous callus that eventually ossifies and bridges the fracture.^1,2^ In aged individuals, a significant decline in SSPC numbers is a key factor underlying impaired fracture healing.^3–7^ Although depending on local microenvironmental cues, the upstream sources of these cues and how they drive SSPC depletion remain poorly defined.

Marrow adiposity increases with age and has been linked to impairments in bone healing, coincident with a decline in SSPC numbers and proliferative capacity.^8–11^ Yet the functional contribution of bone-marrow adipocytes to SSPC regulation remains unclear. Bone marrow Adiponectin^+^ (Adipoq^+^) cells (hereafter BMACs) are mainly Adipoq^+^ adipocytes and their non-lipid-laden adipogenic precursors (MALPs). Although Adipoq-lineage encompasses a subset of marrow mesenchymal progenitors, *Adipoq-Cre* predominantly recombines in adipose-lineage cells with limited direct osteogenic contribution in young adult mice,^12–16^ and is undetectable in periosteal SSPCs that dominate bicortical fracture healing.^15,17^ BMACs have emerged as an active source of marrow-derived signals that shape the bone microenvironment.^12,13,18^ For instance, BMACs support hematopoietic stem cells by secreting stem cell factor (SCF),^19,20^ and they produce factors such as Grem1, RANKL, M-CSF, and SOST, which modulate bone homeostasis.^12,13,16,21–26^ In addition, marrow adipocytes also secrete adipokines, inflammatory cytokines, and lipids, likely influencing the skeletal cells.^27–31^ Importantly, BMACs undergo cellular senescence during aging and other pathological conditions.^32–35^ Cellular senescence refers to a state of stable cell-cycle arrest accompanied by production of senescence-associated secretory phenotypes (SASPs), which are a broad spectrum of proinflammatory cytokines and chemokines that are usually hostile to the stem cell niche and impair tissue homeostasis and regeneration.^36–38^ Despite this progress, it remains unknown whether and how BMAC-derived signals maintain a broader spectrum of SSPCs—including marrow Adipoq^-^ and periosteal progenitors—and whether dysregulated BMAC states causally impair bone repair by impairing the SSPC niche. Moreover, the intrinsic regulatory pathways governing BMAC homeostasis and senescence remain to be elucidated.

Core-binding factor β (Cbfβ) is a co-transcription factor that binds to the Runt domain of RUNX proteins, stabilizing their interaction with the DNA motif 5’‑PuACCPuCA‑3’,^39^ thereby controlling gene programs essential for skeletal development and postnatal bone homeostasis.^40–48^ We previously showed that Cbfβ controls the osteogenic-adipogenic fate decision in Prx1^+^ SSPCs,^47^ yet its role in BMACs remains unexplored. Here, using *Adipoq-Cre*-driven conditional deletion of Cbfβ (CKO) in young adult mice, we discovered that loss of Cbfβ in BMACs induces cellular senescence and a senescence-associated secretory phenotype (SASP). This senescent BMAC program is accompanied by genome-wide reductions in chromatin accessibility, especially at DNA-repair-associated loci, consistent with impaired genome maintenance, and leads to depletion of multiple SSPC subsets, including periosteal populations not recombined by *Adipoq-Cre*. Functionally, these changes culminate in defective bicortical fracture healing. Together, our findings identify BMAC senescence as a previously unrecognized non-cell-autonomous mechanism that governs SSPC maintenance and bone repair, and position the Cbfβ-BMAC axis as an upstream regulator with translational relevance for enhancing fracture healing.

## Results

### Adipoq^+^ cells regulate fracture healing via Cbfβ-dependent signaling

**Fig. 1:**
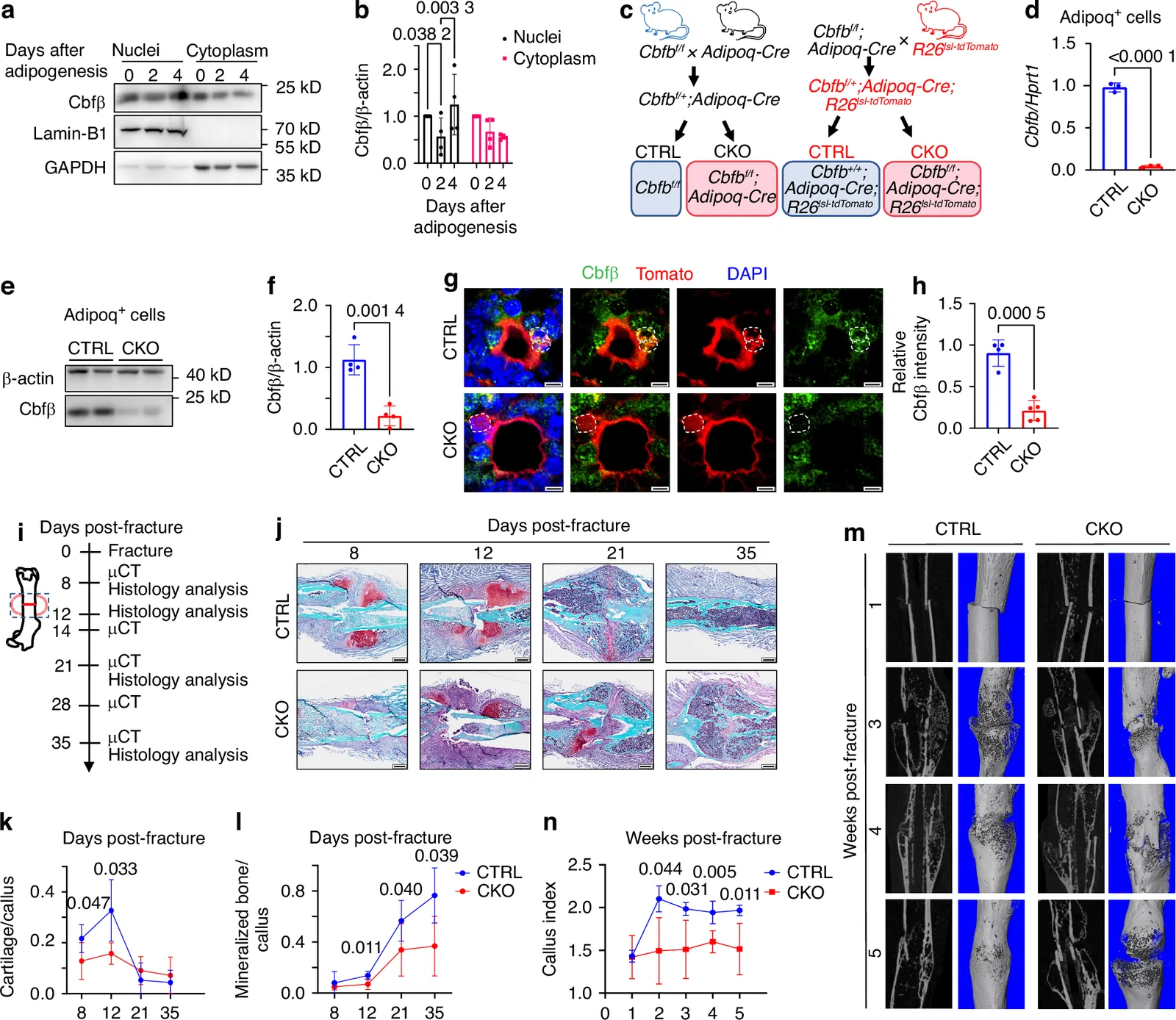
Adipoq^+^ cells regulate fracture healing via Cbfβ-dependent signaling. Western blot of Cbfβ in subcellular fractions during mesenchymal stem cells (MSCs) adipogenic differentiation (**a**) with quantification (**b**). *n* = 4. **c** Schematic of Cbfβ conditional knockout (CKO) mice (*Cbfb*^*f/f*^*;Adipoq-Cre*) and controls (*Cbfb*^*f/f*^), and *tdTomato* lineage-tracing CKO mice (*Cbfb*^*f/f*^*;Adipoq-Cre;tdTomato*) with controls (*Adipoq-Cre;tdTomato*). **d** RT-qPCR confirming *Cbfb* deletion in sorted Adipoq^+^ cells from CKO mice. *n* = 3-4. Western blot of Cbfβ in sorted Adipoq^+^ cells from control and CKO mice (**e**) with quantification (**f**). *n* = 4. Anti-Cbfβ immunofluorescence (IF) in femoral sections from 2-month-old control and CKO mice (**g**) with quantification (**h**). *n* = 4-5. Scale bar: 5 μm. **i** Schematic of femoral fracture model and timeline. Safranin O/fast green staining of callus from control and CKO male mice (**j**); cartilage and mineralized bone volumes quantified in (**k**, **l**). *n* = 4–7. Scale bar: 500 μm. μCT analysis of fracture healing (**m**); callus index quantified in (**n**). *n* = 3-6. Data are presented as mean ± SD; *P*-values: two-tailed unpaired *t*-test (**d**, **f**, **h**, **k**, **l**, **n**) and two-way ANOVA with Fisher’s LSD (**b**)

Bone marrow Adipoq^+^ cells (BMACs) are active components of the skeletal microenvironment and expand during aging as SSPCs decline and bone healing becomes impaired. Given this inverse relationship, we hypothesized that BMACs regulate SSPC maintenance and skeletal regeneration. Cbfβ, a co-transcription factor of the RUNX family involved in osteoblast-adipocyte lineage commitment,^47^ showed stage-dependent expression during mesenchymal stem cells (MSCs) adipogenesis, with an initial decrease followed by up-regulated expression at later stages (Fig.1a, b), suggesting a potential role in BMAC function.

To test this, we generated Adipoq^+^ cell-specific Cbfβ conditional knockout mice (Cbfβ CKO, *Cbfb*^*f/f*^*;Adipoq-Cre*) (Fig. 1c), with age- and sex-matched *Cbfb*^*f/f*^ littermate controls. To verify the specificity of *Adipoq-Cre* activity, we introduced a *tdTomato* reporter allele (*Cbfb*^*f/f*^*;Adipoq-Cre;tdTomato*), using *Adipoq-Cre;tdTomato* as controls (Fig. 1c). PCR genotyping confirmed the presence of *floxed* and wild-type *Cbfb* alleles, *tdTomato*, and *Adipoq-Cre* transgenes (Fig. S1a). BMACs were isolated by FACS (Fig. S1b), and efficient Cbfβ deletion in BMACs was validated by qPCR, western blot, and immunofluorescence (Fig. 1d–h). Unlike *Prx1*-*Cre*-mediated deletion, this model did not affect skeletal development, enabling assessment of postnatal function.

Using a femoral fracture model, we found that Cbfβ deletion markedly impaired bone repair (Fig. 1i). Fracture repair proceeds through sequential phases of inflammation, soft callus formation, hard callus formation, and remodeling.^1,49^ Histological analyses were performed at post-fracture days 8, 12, 21, and 35, and μCT analyses at days 8, 14, 21, 28, and 35 (Fig.1i–n; Fig. S1c–e). In control mice, fracture healing progressed from cartilage callus (days 8–12) to mineralized callus (day 21) and complete bridging (day 35) (Fig. 1j–l). In contrast, CKO mice showed reduced cartilage callus at day 12 (−51.7%), delayed mineralization at day 21 (−40.3%), and persistent nonunion at day 35 (Fig. 1j–l). TRAP staining revealed reduced osteoclast numbers at later stages (days 21 and 35), indicating impaired remodeling (Fig. S1f–h). Consistently, μCT analysis and callus index quantification confirmed severely impaired callus formation in Cbfβ CKO mice (Fig. 1m, n; Fig S1c–e). Together, these findings demonstrate that Cbfβ deletion in Adipoq^+^ cells profoundly impairs bone repair and leads to nonunion, supporting a critical role for BMAC in skeletal regeneration through Cbfβ-dependent signaling.

### Cbfβ in Adipoq^+^ cell maintains SSPC populations

**Fig. 2:**
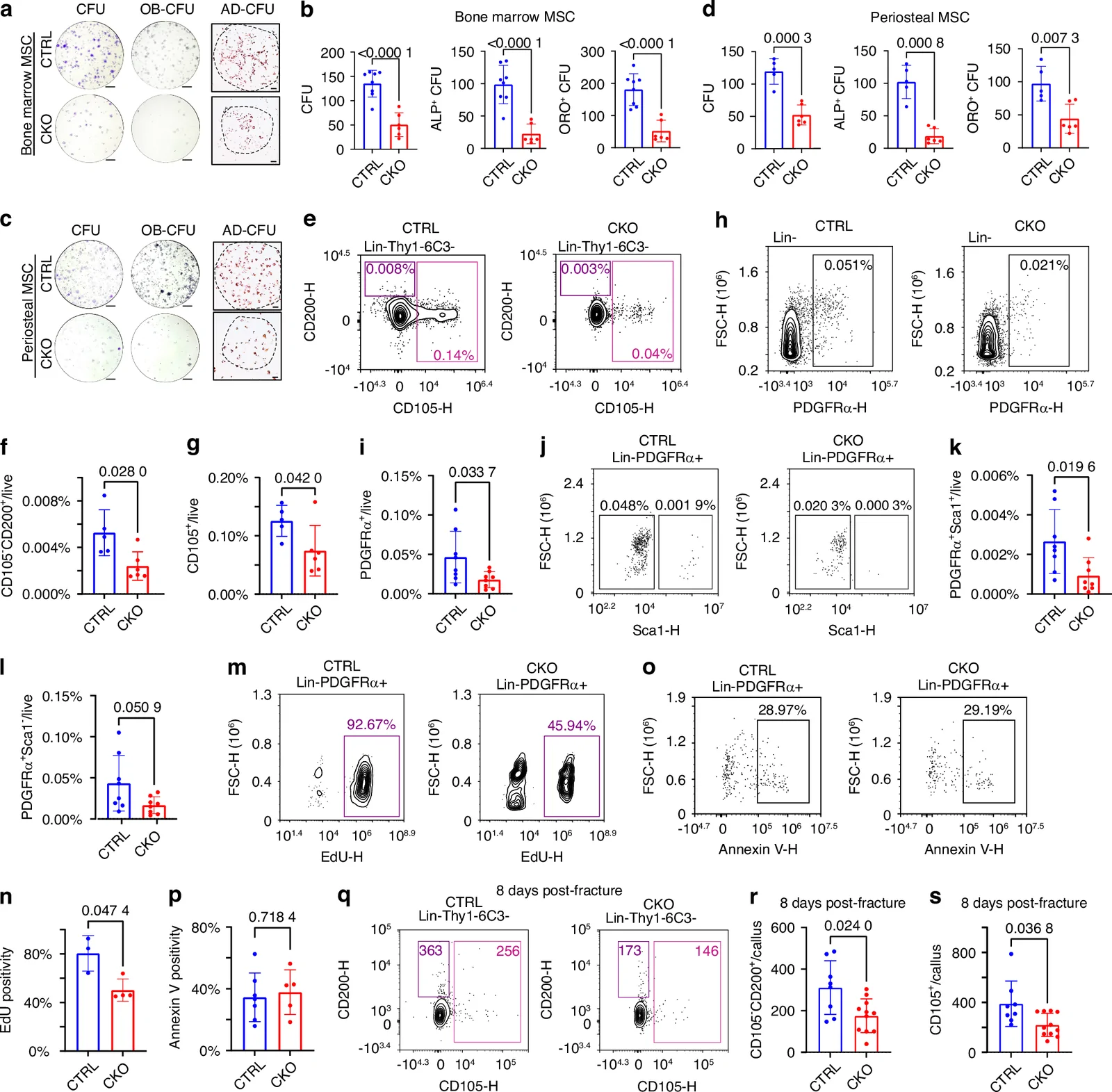
Cbfβ in Adipoq^+^ Cell is essential for SSPC population maintenance. **a**, **b** CFU assay and quantification of bone marrow MSCs from 2-month-old control and Cbfβ CKO mice. *n* = 6–8. **c**, **d** CFU assay and quantification of periosteal MSCs. *n* = 5–6. Total CFU: Crystal Violet; OB-CFU: ALP; AD-CFU: Oil Red O. Scale bars: CFU/OB-CFU, 5 mm; AD-CFU, 50 μm. **e**–**g** Flow cytometry and quantification of CD105^-^CD200^+^ and CD105^+^ SSPCs from whole femora. *n* = 5–6. **h**–**l** Flow cytometry and quantification of PDGFRα^+^ SSPCs from periosteal femoral bone, including PDGFRα^+^Sca1^+^ and PDGFRα^+^Sca1^-^ populations; *n* = 8. **m**, **n** Flow cytometry and quantification of EdU^+^ PDGFRα^+^ SSPCs to assess proliferation; *n* = 3-4. **o**, **p** Flow cytometry analysis and quantification of Annexin V^+^ PDGFRα^+^ SSPCs to assess apoptosis; *n* = 5–7. **q**–**s** Flow cytometry analysis and quantification of CD105^-^CD200^+^ and CD105^+^ SSPCs from callus in fracture model; *n* = 8–11. Data are presented as mean ± SD; *P*-values: two-tailed unpaired *t*-test

SSPCs are essential for bone regeneration. Given the observed defects in bone repair, we first assessed SSPC self-renewal and differentiation capacity using colony-formation (CFU) assays in bone marrow- and periosteum-derived MSCs, which comprise heterogeneous SSPC populations. CFU formation was markedly reduced in both compartments, with total CFU, OB-CFU, and AD-CFU decreased by 62.5%, 77.2%, and 71.2% in bone marrow MSCs, and by 56.2%, 81.7%, and 54.6% in periosteal MSCs (Fig. 2a–d). Lineage tracing further showed that CFU formation in marrow MSCs was reduced in both Adipoq^+^ and Adipoq^−^ populations (Fig. S2a, b), indicating that Cbfβ deficiency in BMACs impairs SSPCs through both intrinsic and non-cell-autonomous effects.

We next quantified defined SSPC subpopulations by flow cytometry, including CD105^-^CD200^+^ SSPCs (CD45^-^CD31^-^Ter119^-^Thy1^-^6C3^-^CD105^-^CD200^+^), CD105^+^ SSPCs (CD45^-^CD31^-^Ter119^-^Thy1^-^6C3^-^CD105^+^, bone cartilage stromal progenitors), PDGFRα^+^ SSPCs (CD45^-^CD31^-^Ter119^-^PDGFRα^+^), PDGFRα^+^Sca1^+^ SSPCs (CD45^-^CD31^-^Ter119^-^PDGFRα^+^Sca1^+^), and PDGFRα^+^Sca1^-^ SSPCs (CD45^-^CD31^-^Ter119^-^PDGFRα^+^Sca1^-^, osteoprogenitor cells).^17,24,50–52^ CD105^-^CD200^+^ and CD105^+^ SSPCs, which possess osteogenic and chondrogenic potential, were reduced by 54.4% and 40.8%, respectively, in Cbfβ CKO mice (Fig. 2e–g, Fig. S2c). PDGFRα^+^SSPCs were reduced by 61.5% (Fig. 2h, i, Fig. S2d), including both PDGFRα^+^Sca1^+^ (trilineage differentiation potential, −65.1%) and PDGFRα^+^Sca1^−^ (osteogenic potential, −61.4%) subsets (Fig. 2j–l, Fig. S2d). EdU incorporation revealed reduced proliferation of PDGFRα^+^ SSPCs, whereas apoptosis remained unchanged (Fig. 2m–p), indicating that SSPC depletion is primarily driven by impaired proliferation. Similar reductions were observed in female mice, confirming that this phenotype is not sex-dependent (Fig. S2e, f). These results demonstrate that Cbfβ deletion in Adipoq^+^ cells profoundly reduce the abundance of multiple SSPC populations. During fracture healing, SSPC expansion was also impaired at the callus sites. At day 8 post-fracture, CD105^−^CD200^+^ and CD105^+^ SSPCs were reduced by 43.7% and 43.6% in CKO mice (Fig. 2q–s). Together, these results demonstrate that Cbfβ deletion in Adipoq^+^ cells compromises both steady-state maintenance and injury-induced expansion of SSPCs, thereby contributing to defective bone repair.

### Cbfβ deficiency in Adipoq^+^ cells reduces bone formation and promotes marrow adiposity and senescence

SSPCs are also essential for bone formation during homeostasis. To investigate whether Cbfβ loss in BMACs affects bone formation, we performed µCT and histological analyses on 2-month-old mice. µCT analysis revealed no significant changes in bone mass in males and a slight increase in females, which is consistent with reduced osteoclastogenesis (Fig. S3a–f). RNA-seq analysis showed downregulation of osteoclast differentiation-related gene signatures, including *Csf1* and *Tnfsf11* (Fig. S3g–h), and CUT&Tag confirmed Cbfβ binding at their promoter regions (Fig. S3i–j), suggesting a direct regulatory role.

**Fig. 3:**
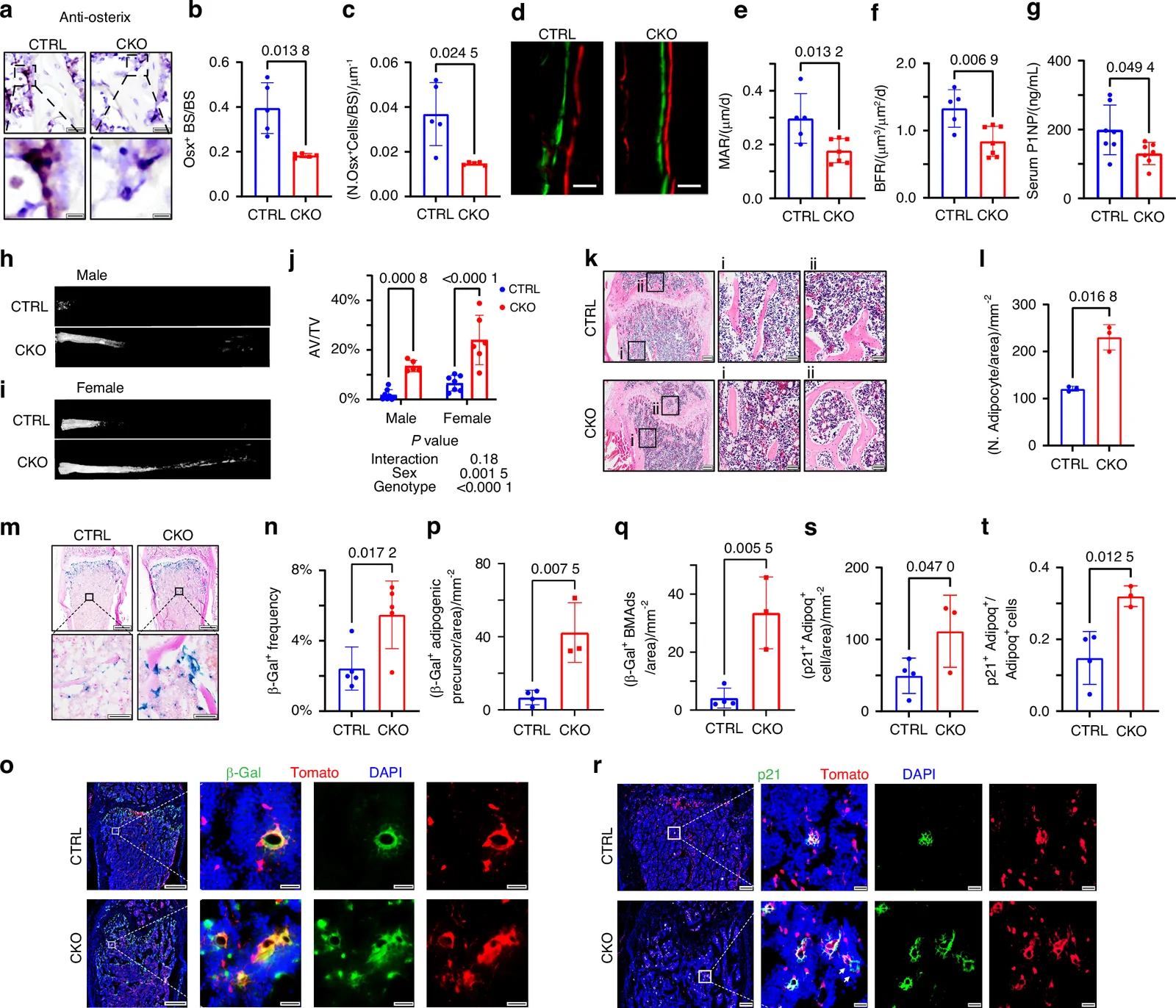
Cbfβ deficiency in Adipoq^+^ cells reduces bone formation and increases marrow Adipoq^+^ cell senescence. Bone histomorphology analysis using femoral bone sections from 2-month-old control and Cbfβ CKO mice: Anti-Osterix (Osx) immunohistochemistry (IHC) in (**a**); Osx^+^.BS/BS and N.Osx^+^/BS quantified in (**b**, **c**). *n* = 5. Scale bars: upper 20 μm; lower 5 μm. Calcein-Alizarin complexone double labeling for bone formation analysis in (**d**); mineral apposition rate (MAR) and bone formation rate (BFR) quantified in (**e**, **f**). *n* = 5–7. Scale bar: 100 μm. **g** ELISA of serum P1NP; *n* = 7. OsO4 staining and μCT analysis of decalcified tibiae showing marrow adiposity in (**h**, **i**); quantification of adipose volume in tissue volume (AV/TV) in (**j**); *n* = 6–7. HE staining (**k**) with quantification of N.Adipocyte/area (**l**). *n* = 3. Scale bars: leftmost 200 μm; others 50 μm. Senescence-associated β-galactosidase (β-Gal) staining (**m**) with quantification of β-Gal^+^ cells in (**n**). *n* = 5. Scale bars: upper 500 μm; lower 50 μm. β-Gal IF staining (**o**) with quantification of β-Gal^+^ adipogenic precursors and BMAds in (**p**, **q**). *n* = 3-4. Scale bars: leftmost 500 μm; others 20 μm. Anti-p21 IF staining (**r**) with quantification of p21^+^Adipoq^+^ cells in (**s**, **t**). *n* = 3–4. Scale bars: leftmost 200 μm; others 20 μm. Data are presented as mean ± SD; *P*-values were determined using two-tailed unpaired *t*-tests (**b**, **c**, **e**, **f**, **g**, **l**, **n**, **p**, **q**, **s**, **t**) and two-way ANOVA with Fisher’s LSD test (**j**)

Despite this, bone formation was also markedly impaired in Cbfβ CKO mice (Fig. 3a–g). Osx^+^ osteoblast density and surface were reduced by 60.1% and 53.6%, respectively (Fig. 3a–c). Dynamic histomorphometry showed decreased mineral apposition rate (MAR,↓40.4%) and bone formation rate (BFR,↓36.9%) (Fig. 3d–f), and serum P1NP levels were reduced by 34.5% (Fig. 3g), indicating diminished osteoblast activity.

Given that Adipoq^+^ cells include both adipogenic precursors and mature adipocytes, we next assessed marrow adiposity. Osmium tetroxide-based μCT revealed a marked increase in marrow fat (7.2-fold in males and 3.6-fold in females; Fig. 3h–j), which was confirmed histologically (Fig. 3k–l). Lineage tracing combined with BODIPY staining further showed an increased proportion of mature adipocytes, accompanied by a concomitant reduction in adipogenic precursors (Fig. S4a–e).

We next examined cellular senescence, given its established role in SSPC dysfunction and impaired bone repair.^53–56^ β-Galactosidase (β-Gal) staining revealed a marked increase in marrow senescence in Cbfβ CKO mice (Fig. 3m, n). Notably, senescent Adipoq^+^ cells (β-Gal^+^tdTomato^+^) were significantly increased, with mature adipocytes and adipogenic precursors elevated by 8.1-fold and 6.3-fold, respectively (Fig. 3o–q). This was further supported by increased p21^+^tdTomato^+^ BMACs (Fig. 3r–t). Importantly, these changes were largely restricted to the marrow compartment. Body weight and peripheral adipose depots [inguinal white adipose tissue (iWAT), epididymal white adipose tissue (eWAT), brown adipose tissue (BAT)] were unchanged (Fig. S4f–i), with no differences in adipocyte size (Fig. S4j, k). Consistently, *Cbfb* expression was higher in marrow adipose tissue than in peripheral fat (Fig. S4l), suggesting a marrow-specific role.

Together, these results demonstrate that Cbfβ loss in Adipoq^+^ cells selectively impairs bone formation while promoting marrow adiposity and senescence, thereby altering the skeletal microenvironment.

### Cbfβ deficiency induces senescence in marrow Adipoq^+^ cells via DNA damage accumulation

**Fig. 4:**
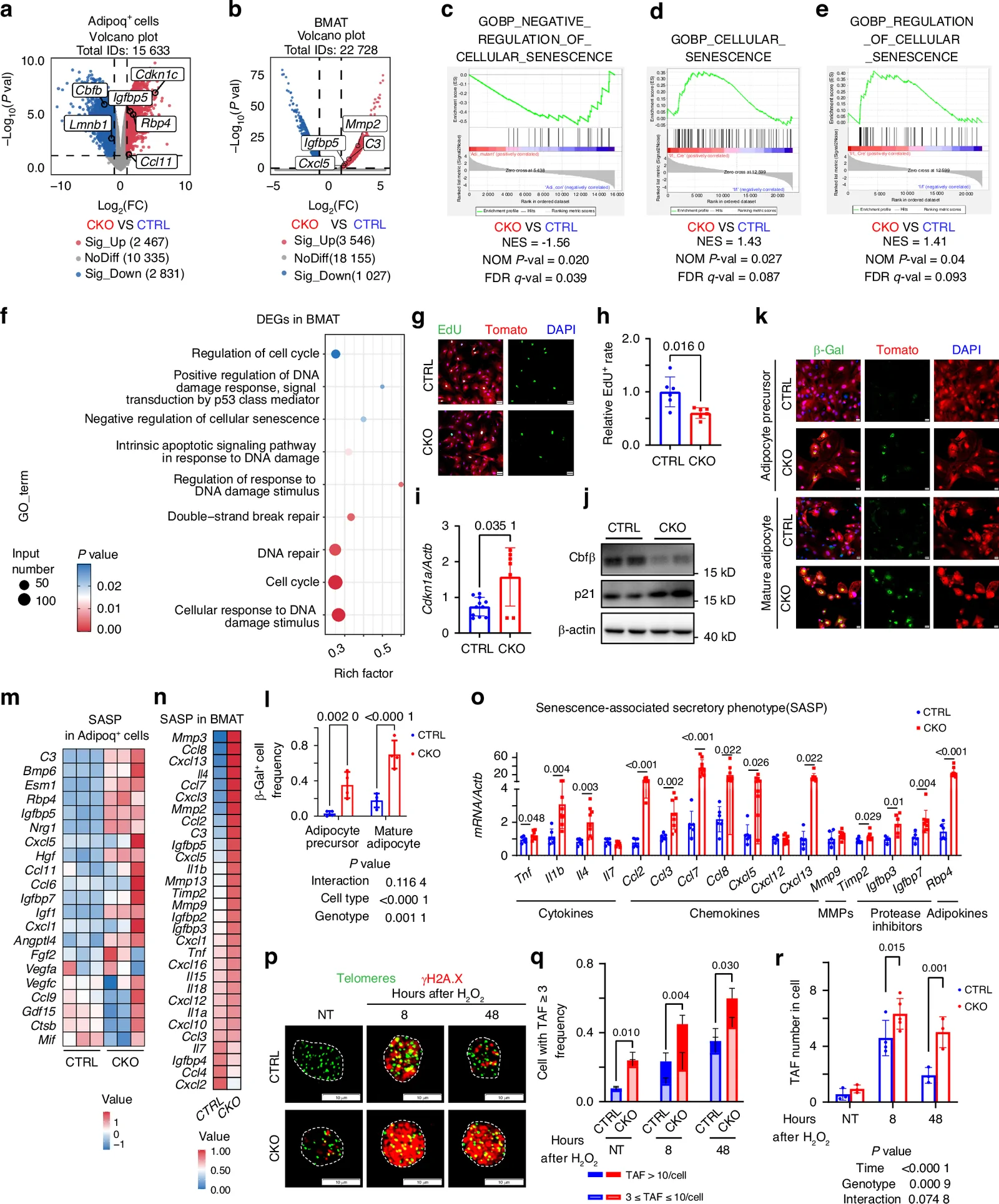
Cbfβ deficiency induces senescence in Adipoq^+^ cells via increasing DNA damage. **a** Volcano plot of differentially expressed genes (DEGs) in RNA-seq of Adipoq^+^ cells sorted based on tdTomato fluorescence from 2-month-old control and Cbfβ CKO mice; *n* = 3. **b** Volcano plot of DEGs in marrow adipose tissue from control and Cbfβ CKO mice; *n* = 1 pooled from 10 mice. **c** GSEA showing downregulation of genes negatively regulating senescence in Cbfβ CKO Adipoq^+^ cells; *n* = 3. **d**, **e** GSEA showing upregulation of senescence-related genes in marrow adipose tissue from CKO mice; *n* = 1 pooled from 10 mice. **f** Bubble plot of GO enrichment for senescence-related DEGs in marrow adipose tissue; *n* = 1 pooled from 10 mice. EdU incorporation in Adipoq^+^ cells (**g**) with quantification of EdU^+^ cells (**h**); *n* = 6. Scale bar: 50 μm. **i** RT-qPCR of *Cdkn1a* mRNA in control and CKO Adipoq^+^ cells; *n* = 7–11. **j** Western blot of p21 protein in control and CKO Adipoq^+^ cells; *n* = 4. β-Gal IF staining of Adipoq^+^ cells (**k**) with quantification of β-Gal^+^ cells (**l**) showing increased senescence in CKO; *n* = 4. Scale bars: 20 μm. **m** RNA-seq heatmap showing upregulation of SASP genes in CKO Adipoq^+^ cells; normalized by Z-score; *n* = 3. **n** RNA-seq heatmap showing upregulation of SASP genes in CKO marrow adipose tissue; normalized by scaling; *n* = 1 pooled from 10 mice. **o** RT-qPCR validation of SASP gene upregulation in CKO marrow adipose tissue; *n* = 6–9. **p**–**r** IF analysis of telomere-associated DNA damage foci (TAF) in Adipoq^+^ cells with/without H_2_O_2_ (**p**). Telomeres: TelC-488; DNA damage: anti-γH2A.X IF. Quantification of cells with ≥3 TAF in (**q**) and TAF number per cell in (**r**); *n* = 3–5. Scale bar: 10 μm. Data are presented as mean ± SD; *P*-values determined by two-tailed unpaired *t*-test (**h**, **i**, **o**), two-way ANOVA (**l**, **q**), or mixed-effects analysis with Fisher’s LSD test (**r**)

To investigate how Cbfβ loss affects Adipoq^+^ cells, we performed RNA sequencing (RNA-seq) on sorted Adipoq^+^ cells and marrow adipose tissue-enriched fractions (Fig. 4; Fig. S1b, S5). Marrow adipose tissue was predominantly localized in the distal tibia and collected through enrichment above a 70 µm filter, and its efficient enrichment was confirmed by increased *Fabp4* expression (Fig. S5a, b).

Transcriptomic analysis revealed extensive changes in both Adipoq^+^ cells (2 467 genes upregulated, 2 831 downregulated) and marrow adipose tissue (3 546 genes upregulated, 1 027 downregulated) (*q* < 0.05, |log_2_(FC)| > 1; Fig. 4a, b). Gene Set Enrichment Analysis (GSEA) showed enrichment of senescence-associated pathways in both datasets (Fig. 4c–e; Fig. S5c–e), and Gene Ontology (GO) analysis highlighted DNA damage response and cell cycle regulation as major affected processes (Fig. 4f; Fig. S5f), consistent with the increased senescence observed in vivo (Fig. 3).

Functionally, Cbfβ-deficient Adipoq^+^ cells exhibited reduced proliferation (−39.6%, EdU; Fig. 4g, h) and increased expression of senescence markers, including *Cdkn1a* (p21) at both mRNA and protein levels (Fig. 4i, j). β-Gal staining further confirmed increased senescence in both adipogenic precursors and mature adipocytes (Fig. 4k, l). In parallel, RNA-seq analysis revealed robust activation of the senescence-associated secretory phenotype (SASP) in both Adipoq^+^ cells and marrow adipose tissue (Fig. 4m, n). This was validated by RT-qPCR, showing upregulation of multiple SASP factors, including IL-1β, CCL2, CCL3, CCL7, CCL8, CXCL5, CXCL13, and IGFBP7 (Fig. 4o).

Given the enrichment of DNA damage-related pathways, we next examined genomic instability. Cbfβ-deficient Adipoq^+^ cells showed a marked increase in γH2A.X foci, particularly telomere-associated foci (TAF),^38,57^ under both basal and oxidative stress conditions (Fig. 4p–r). Cells with ≥3 TAF increased 3.1-fold at baseline, and DNA damage was further exacerbated following H₂O₂ treatment. Severe damage (TAF > 10) and the average number of TAF per cell were also significantly elevated (Fig. 4q, r).

Together, these results indicate that Cbfβ loss leads to DNA damage accumulation and genomic instability in Adipoq^+^ cells, driving cellular senescence and SASP activation, which likely disrupts the SSPC niche.

### Cbfβ in Adipoq^+^ cells regulates bone formation and SSPCs via a non-cell-autonomous mechanism

**Fig. 5:**
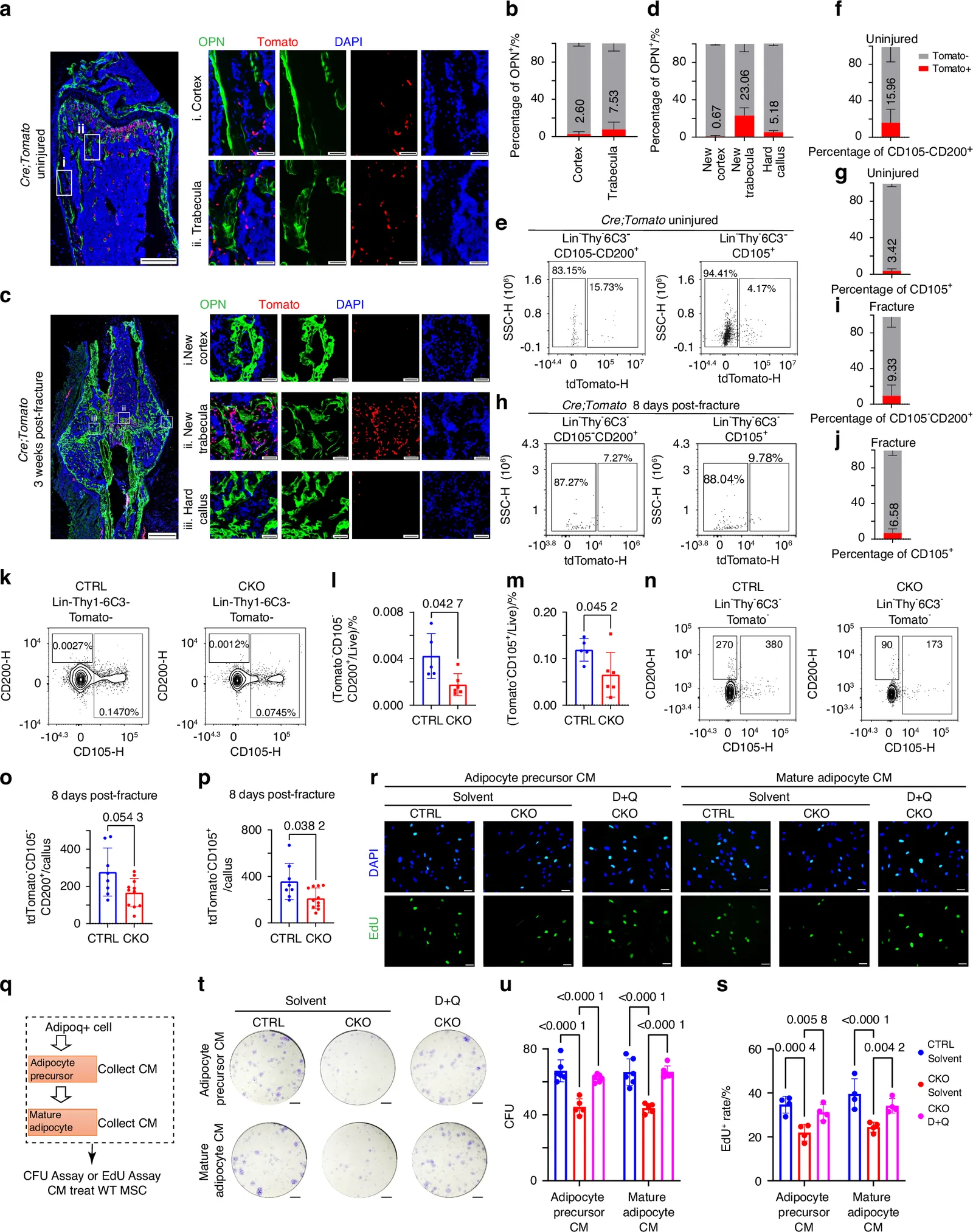
Adipoq^+^ cells regulate bone formation and SSPCs via secretory signaling. Anti-Osteopontin (OPN) IF staining of femoral sections from 2-month-old *Adipoq-Cre;tdTomato (Cre;Tomato)* mice (**a**, **b**) and bone callus 3 weeks post-femoral fracture (**c**, **d**), with quantification of Tomato^+^ cells among OPN^+^ cells (*n* = 3; scale bars: leftmost 1000 µm, others 50 µm). Flow cytometry analysis and quantification of Tomato^+^ percentages in CD105^-^CD200^+^ and CD105^+^ SSPCs from femoral bones during homeostasis (**e**–**g**; *n* = 6) and callus 8 days post-fracture (**h**–**j**; *n* = 9). Flow cytometry analysis of Tomato^-^CD105^-^CD200^+^ and Tomato^-^CD105^+^SSPCs in CKO and control mice during homeostasis (**k**–**m**; *n* = 5–6) and 8 days post-fracture (**n**–**p**; *n* = 8–11). **q** Schematic of conditioned medium (CM) preparation from Adipoq^+^ precursors and mature adipocytes. **r**–**u** EdU incorporation (**r**, **s**, *n* = 4; scale bar=50 µm) and CFU assay (**t**, **u**, *n* = 5–6; scale bar = 5 mm) of marrow MSCs treated with CM from control or CKO Adipoq^+^ precursors and mature adipocytes, with or without Dasatinib+Quercetin (D + Q) with quantification in (**s**, **u**). Data are presented as mean ± SD; *P*-values determined by two-tailed unpaired *t*-tests (**l**, **m**, **o**, **p**) or two-way ANOVA with Fisher’s LSD multiple comparisons (**s**, **u**)

Cellular senescence affects the microenvironment primarily through SASP factor secretion.^6,38,58–60^ To determine whether Cbfβ in Adipoq^+^ cells regulates SSPCs through secretory mechanisms rather than direct differentiation, we performed lineage tracing using tdTomato reporter mice. Adipoq^+^ cells contributed minimally to osteoblast populations under both homeostasis and fracture repair. Only 7.5% of trabecular and 2.6% of cortical OPN^+^ osteoblasts were derived from Adipoq^+^ cells during homeostasis, and their contribution to newly formed bone during fracture repair remained negligible in 2-month-old mice (Fig. 5a–d). This limited contribution of Adipoq^+^ cells to osteoblasts during fracture healing was consistent across age groups (6-month- and 10-month-old mice) and occurred without ectopic activation of *Adipoq*-Cre in the periosteum (Fig. S6a–e), indicating that direct differentiation is unlikely to account for the observed bone phenotype.

Consistently, Adipoq^+^ cells showed limited overlap with SSPC populations, as assessed by flow cytometry. TdTomato^+^ cells comprised only a small fraction of CD105^−^CD200^+^, and CD105^+^ SSPCs under both homeostasis and fracture repair (Fig. 5e–j). Importantly, tdTomato^−^ SSPCs were significantly reduced in Cbfβ CKO mice (Fig. 5k–p), demonstrating that SSPC loss cannot be explained by impaired differentiation of Adipoq^+^ progenitors.

Previous studies have reported that cellular senescence can spread to neighboring cells within the local microenvironment.^35,61^ Consistently, we observed increased senescence in Adipoq^−^ marrow cells in Cbfβ CKO mice, particularly among immune populations such as macrophages and neutrophils (Fig. S7a–g). To determine whether BMAC-intrinsic senescence is sufficient to drive SSPC depletion, independent of secondary senescence spread, we generated inducible Cbfβ CKO mice (*Cbfb*^*f/f*^*;Adipoq-CreERT2*, iCKO). At an early time point following deletion (Fig. S8a–c), SSPC numbers and CFU capacity were already significantly reduced (Fig. S8d–k), while senescence was restricted to Adipoq^+^ cells and not detected in other marrow populations (Fig. S8l–u). These findings indicate that BMAC-intrinsic senescence precedes and is sufficient to drive SSPC depletion.

Despite their confinement to the marrow compartment, Cbfβ-deficient Adipoq^+^ cells also reduced periosteal SSPCs, suggesting long-range signaling. Consistently, BMACs and periosteal PDGFRα^+^ SSPCs were both closely associated with transcortical vascular structures (Fig. S9a, b), and BMACs accumulated in marrow regions adjacent to cortical bone (Fig. S9c). In contrast, cells within the cortical bone compartment exhibited minimal senescence signals (Fig. S9d–i). Together, these spatial observations support a vascular route through which BMAC-derived factors directly influence periosteal SSPCs, rather than secondary senescence propagation.

To directly test the role of secreted factors, we performed conditioned medium (CM) experiments. CM from Cbfβ-deficient BMACs, including both Adipoq^+^ precursors and mature adipocytes, significantly suppressed the proliferation (EdU incorporation) and clonogenic capacity (CFU formation) of both bone marrow and periosteal MSCs, and these effects were rescued by senolytic treatment (D + Q) (Fig. 5q–u; Fig S10a–f). Importantly, CM from Cbfβ CKO Adipoq^+^ cells suppressed IGF-AKT signaling, a critical pathway regulating MSC proliferation and osteogenesis, in both bone marrow and periosteal MSCs (Fig. S10g–j).

We next identified IGFBP7 as a key SASP factor mediating this effect. IGFBP7 expression and secretion were markedly increased in Cbfβ-deficient BMACs (Fig. S11a–d). Recombinant IGFBP7 suppressed MSC proliferation and IGF-AKT signaling, phenocopying the effects of CM; whereas knockdown of IGFBP7 in Cbfβ-deficient BMACs abolished the inhibitory effects of CM (Fig. S11e–p).

Together, these findings demonstrate that Cbfβ maintains SSPC function and bone formation through a non-cell-autonomous mechanism, in part via SASP-associated secretion of IGFBP7 that suppresses IGF-AKT signaling.

### Cbfβ preserves genome stability in BMACs through chromatin accessibility-dependent transcriptional regulation

To investigate how Cbfβ regulates DNA damage and senescence, we performed Cleavage Under Targets and Tagmentation sequencing (CUT&Tag-seq) to map Cbfβ binding and Transposase-Accessible Chromatin sequencing (ATAC-seq) to assess chromatin accessibility.

**Fig. 6:**
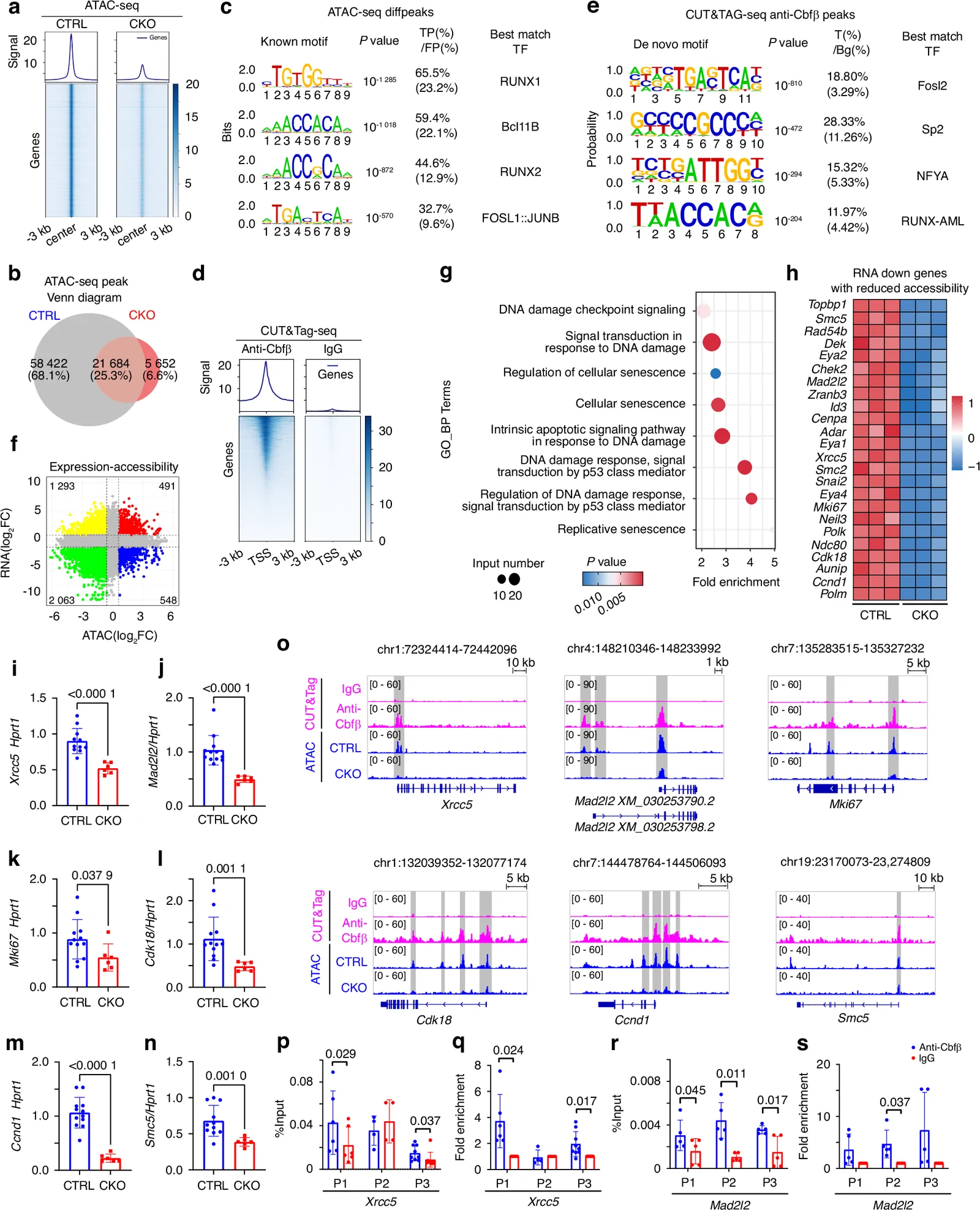
Cbfβ preserves genome stability in BMACs through chromatin accessibility-dependent transcriptional regulation. **a** Genome-wide chromatin accessibility analysis by ATAC-seq using sorted Adipoq^+^ cells; heatmap and average signal centered on peak summits. **b** Venn diagram of accessible chromatin regions in Cbfβ CKO and control cells. **c** Motif enrichment of differentially accessible ATAC-seq peaks; top motifs with *P*-values and corresponding transcription factors shown. **d** Genome-wide Cbfβ binding analysis by CUT&Tag-seq; heatmap and average signal centered on TSS. **e** Motif enrichment of Cbfβ binding sites. **f** Scatter plot of correlation between chromatin accessibility changes (ATAC-seq log₂FC) and gene expression changes (RNA-seq log₂FC). **g** Bubble plot of GO enrichment for genes commonly regulated at chromatin and transcriptomic levels. **h** Heatmap of genome stability-related genes concurrently downregulated in RNA-seq and ATAC-seq; data normalized by Z-score; *n* = 3. **i**–**n** RT-qPCR validation of reduced mRNA expression of indicated genes in Cbfβ CKO Adipoq^+^ cells; *n* = 6-12. **o** IGV tracks of CUT&Tag and ATAC-seq signals at the indicated gene promoters. **p**–**s** ChIP-qPCR confirming Cbfβ occupancy at *Xrcc5* and *Mad2l2* promoters. Data are presented as mean ± SD; *P*-values determined by two-tailed unpaired *t*-test (**i**–**n**) or two-tailed paired *t*-test (**p**–**s**)

ATAC-seq revealed a global reduction in chromatin accessibility upon Cbfβ deletion. Average peak intensity decreased by ~70%, with 72.9% of accessible loci in wild-type cells becoming closed in CKO cells (Fig. 6a, b, Fig S12a, b). Accessibility was reduced across promoters, gene bodies, and intergenic regions, including around transcription start sites (Fig. S12c). Motif analysis showed strong enrichment of RUNX binding sites within regions losing accessibility (RUNX1 motif: 65.5% target, *P* < 10^−1 285^; RUNX2 motif: 44.6% target, *P* < 10^−872^; Fig. 6c), suggesting that Cbfβ maintains chromatin accessibility in cooperation with RUNX factors.

CUT&Tag-seq identified 67 727 Cbfβ-binding sites genome-wide, with strong enrichment at promoter-proximal regions (Fig. 6d; Fig. S12d, e). These regions were also enriched for RUNX motifs (11.97% target, *P* = 10^-204^; Fig. 6e), and chromatin accessibility at Cbfβ-bound loci was markedly reduced following Cbfβ deletion (Fig. S12f–g), indicating a direct role in maintaining open chromatin at target genes.

Integration of ATAC-seq and RNA-seq revealed that 72.9% of downregulated genes exhibited reduced chromatin accessibility, whereas a smaller fraction of upregulated genes showed increased accessibility (Fig. 6f; Fig. S12h, i), indicating that Cbfβ primarily promotes gene expression through maintaining chromatin openness. These genes were significantly enriched in DNA damage response and cell cycle regulation pathways (Fig. 6g, h). Key genome stability-related genes, including *Xrcc5*, *Mad2l2*, *Mki67*, *Cdk18*, *Ccnd1*, and *Smc5*, were significantly downregulated, as confirmed by RT-qPCR and RNA-seq analysis (Fig. 6i–n; Fig. S12j).

Combined ATAC-seq and CUT&Tag-seq analyses showed co-localization of Cbfβ binding with accessible chromatin regions at these target genes (Fig. 6o), and ChIP-qPCR further confirmed direct binding at their promoters (Fig. 6p–s). Analysis of published RUNX2 ChIP-seq data revealed strong RUNX2 occupancy at these loci (Fig. S12k). And CUT&Tag-qPCR further demonstrated that Cbfβ deletion markedly reduced RUNX2 occupancy at these loci (Fig. S12l, m), supporting a RUNX-dependent mechanism. Notably, short-term deletion of Cbfβ in vitro using Ade-Cre reduced chromatin accessibility prior to overt senescence, indicating that chromatin alterations are an early and direct effect of Cbfβ loss (Fig. S13a–e).

Together, these results demonstrate that Cbfβ maintains chromatin accessibility at genome stability-related genes, thereby preserving genomic integrity and preventing senescence in BMACs.

### Senolytic therapy rescued fracture healing defects and SSPC number in Cbfβ CKO mice

To determine whether senescence mediates SSPC loss and impaired bone repair in Cbfβ CKO mice, we tested whether pharmacological clearance of senescent cells could rescue these defects. Mice were treated with the senolytic combination dasatinib and quercetin (D + Q)^62–64^ (Fig. S14a). β-Gal staining confirmed increased senescence in Cbfβ CKO mice, which was markedly reduced by D + Q treatment (Fig. S14b–e). β-Gal/Adiponectin/Perilipin-1 co-staining confirmed that D + Q treatment alleviated senescence in both Adipoq^+^ stromal cells and mature adipocytes (Fig. S14f–h), although marrow adiposity remained unchanged (Fig. S14i, j). Importantly, D + Q treatment significantly improved fracture healing, as evidenced by increased callus formation and mineralized bone volume at day 28 post-fracture (Fig. S14k–n).

Consistent with this, multiple SSPC populations, including CD105^-^CD200^+^ SSPCs, CD105^+^ SSPCs, PDGFRα^+^ SSPCs, PDGFRα^+^Sca1^+^ SSPCs, and PDGFRα^+^Sca1^-^ SSPCs, were significantly reduced in Cbfβ CKO mice and partially restored following D + Q treatment (Fig. S14o–v). Notably, the abundance of senescent (β-Gal^+^) Adipoq^+^ cells was strongly negatively correlated with PDGFRα^+^ SSPC numbers (R² = 0.773, *P* < 0.001; Fig. S14w).

Together, these results demonstrate that senescence is a key driver of SSPC depletion and impaired fracture healing in Cbfβ CKO mice.

### Age-associated decline of Cbfβ in BMACs contributes to impaired skeletal regeneration

**Fig. 7:**
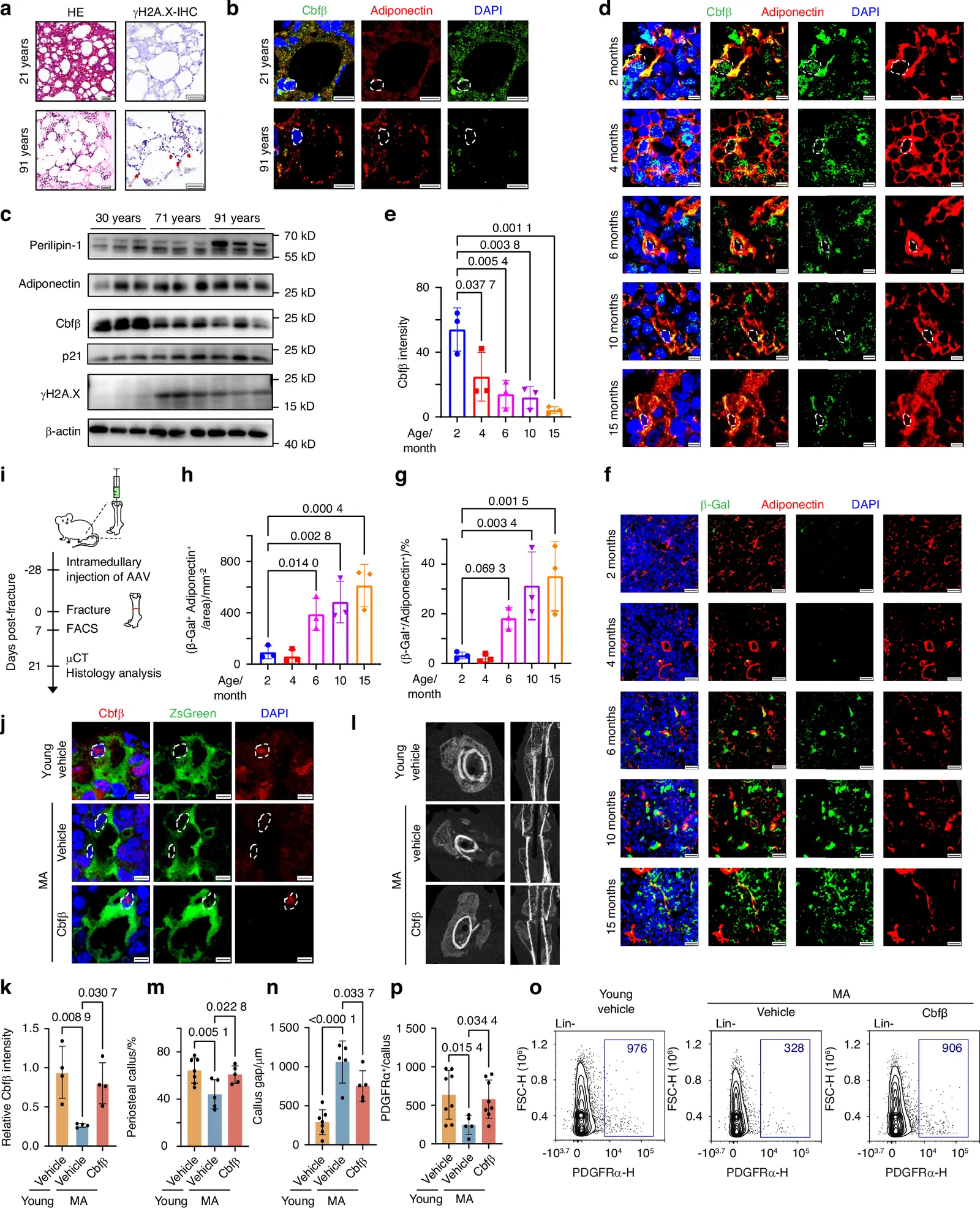
AAV-mediated Cbfβ restored SSPCs and promoted bone healing in middle-aged mice. Representative HE staining and anti-γH2A.X IHC (**a**; scale bars: 100 μm (HE) or 50 μm (IF)), and anti-Cbfβ/Adiponectin IF (**b**; scale bar: 10 μm) of human marrow adipose tissue (hMAT) from 21- and 91-year-old males; *n* = 1 per age. Red arrows indicate γH2A.X^+^ adipocytes. **c** Western blot of Cbfβ and senescence-associated proteins in human MSCs from donors of different ages after adipogenic differentiation. Anti-Cbfβ IF (**d**, **e**; *n* = 3; scale bar: 5 μm) and β-gal staining (**f**–**h**; *n* = 3; scale bar: 20 μm) were used to assess BMACs from mice aged 2, 4, 6, 10, and 15 months; anti-Adiponectin IF-labeled BMACs. **i** Schematic of AAV-mediated Cbfβ rescue in Adipoq^+^ cells and experimental timeline. **j**, **k** Anti-Cbfβ IF and quantification in ZsGreen-labeled Adipoq^+^ cells from young and middle-aged (MA) mice post-AAV injection; *n* = 4. Scale bars: 5 μm. **l**–**n** μCT analysis of femoral fracture callus in young and MA mice with/without Cbfβ restoration; *n* = 5–7. **o**, **p** Flow cytometry analysis of PDGFRα^+^ SSPCs in bone callus; *n* = 5–8. Data are presented as mean ± SD; *P*-values determined by one-way ANOVA with Fisher’s LSD multiple comparisons

To assess the relevance of the Cbfβ-BMAC axis in aging, we analyzed both human and mouse samples. Histological analysis of human marrow adipose tissue (hMAT) revealed increased γH2A.X^+^ senescent adipocytes and reduced Cbfβ expression in aged individuals compared with young donors (Fig. 7a, b). Similarly, in human MSCs undergoing adipogenesis, Cbfβ levels declined with donor age, accompanied by increased senescence-associated markers (Fig. 7c). Consistent changes were observed in mice, where Cbfβ expression in Adiponectin^+^ BMACs progressively decreased with age, while senescent BMACs accumulated starting at 6 months (Fig. 7d–h).

To determine whether restoring Cbfβ expression in Adipoq^+^ cells could reverse aging-associated defects, we employed an AAV-mediated Cbfβ overexpression strategy (Fig. 7i). Successful targeting of Adipoq^+^ cells was confirmed by ZsGreen labeling and increased Cbfβ expression following AAV administration (Fig. 7j, k). Notably, Cbfβ overexpression significantly improved fracture healing in middle-aged mice, as evidenced by increased callus formation and mineralized bone volume in μCT analysis (Fig. 7l–n). In parallel, flow cytometry analysis revealed that AAV-mediated Cbfβ restoration significantly increased the frequency of Lin^−^ PDGFRα^+^ SSPCs (Fig. 7o, p). Together, these findings demonstrate that age-associated decline of Cbfβ in BMACs contributes to SSPC depletion and impaired bone repair, and that restoring this axis partially rescues skeletal regeneration during aging.

## Discussion

Delayed fracture healing and nonunion are major complications of skeletal aging, yet the upstream niche lesions that drive age-associated SSPC exhaustion remain incompletely defined.^4,6,7^ Here, we identify bone marrow Adipoq^+^ cells (BMACs) as a previously underappreciated regulator of skeletal stem/progenitor cell maintenance and bone repair. Our data show that Cbfβ safeguards BMAC homeostasis by maintaining chromatin accessibility and genome stability, thereby preventing premature senescence and SASP activation. Loss of Cbfβ in Adipoq^+^ cells induces BMAC senescence, disrupts the marrow microenvironment, reduces multiple SSPC populations in both marrow and periosteum, and ultimately leads to defective fracture healing. These findings establish the Cbfβ-BMAC axis as a key upstream regulator of skeletal regeneration.

A major conceptual advance of this study is the demonstration that BMACs regulate SSPCs predominantly through a non-cell-autonomous, secretory mechanism. Although Adipoq-lineage cells include mature marrow adipocytes, their adipogenic precursors, and a subset of marrow mesenchymal stromal cells,^18,19,26^ lineage-tracing analyses revealed minimal direct contribution of Adipoq^+^ cells to osteoblasts during skeletal homeostasis in young adult mice, consistent with previous findings.^12–16^ Importantly, Adipoq^+^ cells also showed negligible contribution to fracture repair in both young and older mice in our and published models.^15,17^ Moreover, *Adipoq-Cre* activity was not detected in periosteal stromal cells during fracture healing, whereas periosteal SSPCs were nonetheless significantly reduced in CKO mice. Together with the reduction of tdTomato^−^ SSPCs, the inducible Cbfβ-iCKO model, and the conditioned-medium experiments, these observations strongly support a non-cell-autonomous niche function of BMACs. While senescence propagation to neighboring cells was observed, consistent with previous reports,^65,66^ inducible deletion experiments indicate that BMAC-intrinsic senescence precedes and is sufficient to drive SSPC depletion, identifying BMAC dysfunction as an upstream trigger of niche deterioration.

The effect on periosteal SSPCs is particularly notable given the marrow-restricted localization of Adipoq^+^ cells. Our data implicate a model in which BMAC-derived factors may reach periosteal target cells through local transcortical vascular routes,^67^ thereby extending marrow-derived niche signals beyond the marrow cavity. This interpretation is supported by the close spatial association among BMACs, cortical vessels, and periosteal PDGFRα^+^ SSPCs, together with the minimal senescence detected in cortical cells and the direct inhibitory effects of CKO BMAC-conditioned medium on periosteal MSCs. Although this model requires further direct validation, it provides a plausible explanation for how a marrow-restricted senescent niche can influence periosteal progenitors that are essential for bicortical fracture healing.

Mechanistically, our study places Cbfβ upstream of BMAC senescence by linking it to chromatin accessibility and genome maintenance. Multi-omics integration revealed that Cbfβ loss leads to widespread chromatin closure, particularly at loci involved in DNA repair (*Xrcc5* and *Mad2l2*), cell-cycle progression (*Ccnd1* and *Cdk18*), and chromosomal integrity (*Mki67* and *Smc5*).^68–79^ These alterations precede overt senescence after short-term Cbfβ deletion, indicating that chromatin collapse is likely an early event rather than merely a secondary consequence of senescence. The strong enrichment of RUNX motifs within Cbfβ-dependent accessible regions, together with reduced RUNX occupancy at DNA-repair target loci in Cbfβ-deficient cells, supports a previously underappreciated role for the Cbfβ-RUNX axis in maintaining chromatin accessibility and DNA-repair gene expression in BMACs. Thus, beyond its canonical role as a transcriptional cofactor, Cbfβ functions as a safeguard of the epigenetic landscape required for BMAC integrity. Consistent with this interpretation, RUNX proteins are known to act as pioneer factors, facilitating chromatin accessibility by binding closed regions, such as RUNX1 in hematopoietic cells and RUNX3 in cytotoxic T lymphocytes.^80,81^ RUNX proteins likely interact with chromatin remodeling complexes (e.g., SWI/SNF) to regulate target genes.^81^ Thus, Cbfβ may stabilize RUNX function or directly engage with chromatin remodeling complexes to exert similar effects. The precise chromatin-remodeling partners involved remain to be defined, but our data establish a direct mechanistic link between intrinsic epigenetic instability in BMACs and extrinsic failure of SSPC maintenance.

Our data further indicate that the deleterious effect of senescent BMACs is mediated, at least in part, through SASP-associated secretory signaling, which is known to disrupt niche homeostasis.^36–38^ Conditioned medium from Cbfβ-deficient BMACs suppressed MSC proliferation and clonogenicity, effects that were reversed by senolytic treatment. Among candidate mediators, IGFBP7, a known antagonist of IGF,^82–84^ emerged as a key effector, recapitulating the inhibitory effects on IGF-AKT signaling^85^ and MSC function, while its knockdown attenuated these effects. Although IGFBP7 is unlikely to act alone, given the complexity of the SASP network,^12,13,16,21–31^ these findings highlight a functional link between BMAC senescence and disruption of SSPC-supportive signaling networks.

Importantly, our findings suggest that BMAC senescence may represent an early and targetable driver of skeletal aging. In both human and mouse samples, aging was associated with reduced Cbfβ expression and increased senescence markers in marrow adipose cells. Conversely, restoring Cbfβ expression in Adipoq^+^ cells by AAV delivery in middle-aged mice increased SSPC abundance and improved fracture repair, while senolytic treatment partially rescued the CKO phenotype without normalizing marrow adiposity. Together, these findings suggest that the functional state of BMACs (“quality”) may be more critical than their absolute abundance (“quantity”) in determining their impact on skeletal regeneration, providing a potential explanation for the heterogeneous relationship between marrow adiposity and bone health.^86–88^ Our study, therefore, supports the idea that preserving or rejuvenating BMAC function may represent a more rational therapeutic strategy for aging-associated fracture repair defects.

Several limitations should be acknowledged. First, Adipoq-Cre targets both mature marrow adipocytes and their precursors, precluding precise dissection of their relative contributions. Our data suggest that both populations participate, with mature adipocytes likely contributing more prominently because of their numerical expansion during aging and in CKO mice. Second, the proposed transcortical vascular signaling mechanism remains to be directly demonstrated. Third, while IGFBP7 was identified as a major mediator, it likely represents only part of the broader SASP program induced by Cbfβ loss. Finally, although our chromatin datasets strongly support a role for Cbfβ in maintaining chromatin accessibility, the precise molecular interactions between Cbfβ/RUNX complexes and chromatin-remodeling machinery remain to be fully elucidated.

In summary, this study identifies BMAC senescence as a previously unrecognized upstream niche mechanism controlling SSPC maintenance and fracture repair. By preserving chromatin accessibility and genome stability, Cbfβ safeguards BMAC function and prevents the formation of a senescent, inhibitory microenvironment. These findings redefine marrow adipose-lineage cells as active regulators of skeletal regeneration and nominate the Cbfβ-BMAC axis as a promising therapeutic target for enhancing bone repair during aging.

## Materials and methods

### Generation of mice model

*Cbfb*^*f/f*^ (B6.129P2-Cbfbtm1Itan/J)*, Adipoq-Cre* (B6.FVB-Tg(Adipoq-cre)1Evdr/J), and *R26*^*tdTomato*^ (B6.Cg-Gt(ROSA)26Sortm9(CAG-tdTomato)Hze/J) mice were obtained from Jackson Laboratory. To generate conditional knockout mice, *Cbfb*^*f/f*^ mice, *Adipoq-Cre*, and tdTomato reporter mice were intercrossed, first generating heterozygous offspring, which were subsequently bred to obtain homozygous conditional knockout mice. Littermate *Cbfb*^*f/f*^mice served as controls for *Cbfb*^*f/f*^*;Adipoq-Cre* mice, while *Adipoq-Cre;tdTomato* mice served as controls for *Cbfb*^*f/f*^*;Adipoq-Cre;tdTomato* mice. Genotyping was performed by PCR using genomic DNA extracted from tail biopsies. All mice were housed in an SPF-level animal facility at Zhejiang University under a 12-h light/dark cycle with ad libitum access to standard chow and water. Mice were housed five per cage. All animal procedures were approved by the Institutional Animal Care and Use Committee (IACUC) of Zhejiang University and conducted following the Guide for the Care and Use of Laboratory Animals.

### Generation of the fracture healing model

As previously described, femoral fractures were induced in 8-week-old mice.^89^ Briefly, the femur and patella were surgically exposed, and a 25-gauge syringe needle was inserted parallel to the long axis of the femur through the patellar groove into the marrow cavity. The needle was removed, and a transverse mid-diaphyseal femoral fracture was created using a 0.5-mm Gigli saw. A blunt 25-gauge needle was reinserted through the fracture site into the marrow cavity to stabilize the injury. The injured femur was collected at designated time points for further analysis.

### D + Q senolytic treatment

For the senolytic treatment, 8-week-old mice were administered a combination of Dasatinib (5 mg/kg, Aladdin, CAS No. 302962-49-8) and Quercetin (50 mg/kg, Aladdin, CAS No. 117-39-5) via weekly oral gavage, following established protocols.^53^ The senolytic compounds were prepared in a sterile 1% sodium carboxymethyl cellulose (CMC-Na) solution.

## Supplementary information

BR Supplemental information file-revised

Supplementary Table1

Supplementary Table2

Supplementary Table3

Supplementary Table4

Supplementary Table5

Supplementary Table6

Supplementary Table7

Supplementary Table8

## Data Availability

All data are available in the main text or the supplementary materials.
