# Supplementary material for: Youthfulness of marrow Adipoq+ cells maintained by Cbfβ facilitates stem cell-based bone repair
Source: Bone Res. 2026 Aug 10;14:82. doi: 10.1038/s41413-026-00568-8 (PMC13458481; doi:10.1038/s41413-026-00568-8)
Supplement: Supplementary file 1 [file 41413_2026_568_MOESM1_ESM.docx]

**Supplemental Data and Information**

**Figures and Figure legends**

**Figure S1 Adipoq^+^ Cells Regulate Fracture Healing via Cbfβ-dependent Signaling**

(a) PCR genotyping of mice using tail DNA. Primers detect *Cbfb-WT/f* (~275/400 bp), *Adipoq-Cre* (~220 bp), and *tdTomato-WT/f* (~290/200 bp). (b) Schematic of marrow Adiponectin-expressing (Adipoq^+^) cell isolation. (c-e) μCT images of control (*Cbfb^f/f^*) and Cbfβ CKO (*Cbfb^f/f^;Adipoq-Cre*) female femoral fracture callus 4 weeks post-fracture (c) with quantification of periosteal callus proportion and callus gap (d,e); n=3. (f-h) TRAP staining of bone callus from control and Cbfβ CKO mice (f) with quantification of Oc.S/BS (g) and N.Oc/BS (h); n=3-4. Scale bars: 500 μm. Data are presented as mean ± SD; p-values determined by two-tailed unpaired *t*-tests.

**Figure S2 Cbfβ-deletion Decreased Adipoq⁻ Marrow SSPCs and Flow Cytometric Gating Strategy for SSPCs**

(a-b) Representative Tomato^-^ CFU colonies from bone marrow MSCs of two-month-old control (*Adipoq-Cre;tdTomato*) and Cbfβ CKO (*Cbfb^f/f^;Adipoq-Cre;tdTomato*) mice; quantification in (b); n=6. Scale bar: 500 μm. (c) Schematic of flow cytometric gating for CD105^-^CD200^+^ and CD105^+^ SSPCs. (d) Schematic of flow cytometric gating for PDGFRα^+^ SSPCs, PDGFRα^+^Sca1^+^ SSPCs, and PDGFRα^+^Sca1^-^ SSPCs. (e-f) Flow cytometry and quantification of PDGFRα^+^ SSPCs from periosteal femoral bone of two-month-old control and Cbfβ CKO female mice; n=3. Data are presented as mean ± SD; p-values determined by two-tailed unpaired *t*-test (f) or two-way ANOVA with Fisher’s LSD multiple comparisons (b).

**Figure S3 Cbfβ Deletion Decreases Bone Resorption but Does Not Influence Bone Mass.**

(a-b) Representative μCT images and quantification of tibiae from two-month-old control and Cbfβ CKO female and male mice. n=6-7. (c-e) TRAP staining of femoral sections from control and Cbfβ CKO mice (c) with quantification of Oc.S/BS (d) and N.Oc/BS (e); n=6-7. Scale bars: first row panels, 200 μm; others, 100 μm. (f) Serum CTX1 levels measured by ELISA; n=7. (g) Gene Set Enrichment Analysis (GSEA) showing downregulation of osteoclast differentiation genes in Cbfβ CKO Adipoq^+^ cells compared to controls; n=3. (h) Heatmap of gene expression for osteoclast differentiation; normalized by Z-score; n=3. (i-j) IGV tracks of CUT&Tag-seq signals at promoters of indicated genes. Data are presented as mean ± SD; statistical significance determined by two-tailed unpaired t-test (d-f) or two-way ANOVA with Fisher’s LSD multiple comparisons (b).

**Figure S4 Cbfβ Deletion Increases Marrow Adiposity but Does Not Influence Peripheral Adipose Tissue.**

(a-e) Representative BODIPY lipid staining and quantification of Adipoq^+^ cells in femoral sections from two-month-old control and Cbfβ CKO mice. BODIPY^+^tdTomato^+^ cells indicate bone marrow adipocytes (BMAds), BODIPY^-^tdTomato^+^ cells indicate adipogenic precursors; n=4-5. Scale bars: leftmost panels, 200 μm; others, 50 μm. (f) Body weight of control and Cbfβ CKO male mice from 1 to 6 months; n=6-8. (g-i) Weight of iWAT, eWAT, and BAT in two-month-old control and Cbfβ CKO male mice; n=4-8. (j-k) HE staining of iWAT sections from two-month-old control and CKO mice; quantification of adipocyte size in iWAT; n=3-4. Scale bar: 50 μm. (l) RT-qPCR of *Cbfb* mRNA in BMAT, iWAT, eWAT, and BAT; n=6-8. Data are presented as mean ± SD; statistical significance determined by two-tailed unpaired *t*-test (b-i, k) or one-way ANOVA with Fisher’s LSD multiple comparisons (l).

**Figure S5** **Cbfβ Deficiency Induces Senescence in Adipoq^+^ Cells**

(a) Schematic of BMAT (bone marrow adipose tissue)-enriched fraction collection. (b) RT-qPCR confirming efficient BMAT enrichment, indicated by higher *Fabp4* mRNA expression compared to bone marrow; n=3. (c-d) GSEA showing downregulation of cell cycle-related genes in Cbfβ CKO Adipoq^+^ cells; n=3. (e) GSEA showing downregulation of double-strand break repair genes in Cbfβ CKO Adipoq^+^ cells; n=3. (f) Bubble plot of GO enrichment for downregulated genes in Cbfβ CKO Adipoq^+^ cells, annotated to DNA damage repair; n=3. Data are presented as mean ± SD; statistical significance was determined by a two-tailed unpaired *t*-test.

**Figure S6 Adipoq⁺ Cells Minimally Contribute to Bone Regeneration via Differentiation.**

(a) Representative lineage-tracing images of Adipoq^+^ cells in callus sections from two-month-old *Adipoq-Cre;tdTomato (Cre;Tomato)*mice, 12 days post-fracture; scale bar, 50 μm. (b) Anti-OPN immunofluorescence (IF) of fracture callus sections from six-month-old *Cre;Tomato* mice, 4 weeks post-femoral fracture. (c) Quantification of Tomato^+^ cells among OPN^+^ cells in (b); n=3. (d) Anti-OPN IF of fracture callus sections from 10-month-old mice injected with AAV carrying Adipoq promoter-driven ZsGreen, 3 weeks post-fracture. (e) Quantification of ZsGreen^+^ cells among OPN^+^ cells in (d); n=3. Scale bars in (b) and (d): leftmost panels, 500 μm; others, 50 μm. Data are presented as mean ± SD.

**Figure S7 Cbfβ Deficiency Induces Senescence in Bone Marrow Cells Beyond the Adipoq⁺ Lineage.**

(a) Quantification of β-Galactosidase (β-Gal) Adipoq-lineage-negative cells in femoral sections from two-month-old control and Cbfβ CKO mice; n=3-4. (b-d) Anti-F4/80 IF co-stained with β-Gal staining in femoral sections from two-month-old control and Cbfβ CKO mice (b), with quantification of β-Gal^+^F4/80^+^ proportion (c) and cell number (d); n=3. Scale bar: 20 μm. (e-g) Anti-MPO IF co-stained with β-Gal in femoral sections from two-month-old control and Cbfβ CKO mice (e), with quantification of β-Gal^+^ MPO^+^ proportion (f) and cell number (g); n=4. Scale bar: 10 μm. Data are presented as mean ± SD; p-values determined by two-tailed unpaired *t*-tests.

**Figure S8 Short-term loss of Cbfβ in Adipoq^+^ cells Promotes Adipocyte Senescence and Impairs Skeletal Stem Cell Function.**

(a) Schematic of Tamoxifen-induced Cbfβ conditional knockout (iCKO) mouse model and experimental timeline. (b-c) Anti-Cbfβ IF in femoral sections from control (*Cbfb^+/+^;Adipoq-CreERT2;tdTomato*, +TAM) and Cbfβ iCKO (*Cbfb^f/f^;Adipoq-CreERT2;tdTomato*, +TAM) mice (b) with quantification (c); n=3. Scale bar, 5 μm. (d-h) CFU assays and quantification of marrow (e, f) and periosteal (g, h) stem cells isolated from control and Cbfβ iCKO mice. n=4. Scale bar, 5 mm. (i-k) IF image (i) and quantification of PDGFRα^+^ cells in periosteum (j) and bone marrow (k) of control and Cbfβ iCKO mice; bone and periosteum outlined with dashed lines, * indicates trabecular bone; n=3-4. Scale bar, 50 μm. (l-o) β-Gal staining of femoral sections in (l); quantification of β-Gal^+^Tomato^+^ adipogenic precursors, β-Gal^+^Tomato^+^ BMAds, and β-Gal^+^Tomato^-^ cells in (m-o); n=3. Scale bars: leftmost panels, 500 μm; others, 20 μm. (p-r) Anti-F4/80 IF co-stained with β-Gal in femoral sections in (p); quantification of β-Gal^+^ F4/80^+^ cell number and proportion in (q, r); n=3. Scale bar, 20 μm. (s-u) Anti-MPO IF co-stained with β-Gal in femoral sections in (s); quantification of β-Gal^+^MPO^+^ proportion and number in (t, u); n=4. Scale bar, 20 μm. Data are presented as mean ± SD; p-values determined by two-tailed unpaired *t*-tests.

**Figure S9 BMACs and periosteal SSPCs Communicate Directly via Transcortical Vessels.**

(a) Representative IF of anti-PDGFRα and anti-CD31 in tibial sections, showing periosteal SSPCs adjacent to transcortical vessels; scale bar, 100 μm. (b) Representative anti-CD31 IF of femoral sections from *Cre;Tomato* mice, showing BMACs surrounding CD31^+^ vessels; scale bar, 20 μm. (c) Tomato fluorescence highlighting BMACs adjacent to cortical bone; scale bar, 50 μm. (d-f) Representative β-Gal staining of femoral sections from control and Cbfβ CKO mice (d; scale bar:100 μm) and quantification of β-Gal^+^ cell proportion in cortical bone (e) and β-Gal^+^ cell number per cortical bone area (f); n=3-4. (g-i) Representative β-Gal staining of femoral sections from control and Tamoxifen-induced Cbfβ iCKO mice (g; scale bar=100 μm) and quantification of β-Gal^+^ cell proportion in cortical bone (h) and β-Gal^+^ cell number per cortical bone area (i); n=3. Data are presented as mean ± SD; p-values determined by two-tailed unpaired *t*-tests.

**Figure S10 CKO BMACs Suppress Skeletal Stem Cell Proliferation through Senescence-Associated Secretory Factors.**

(a) β-Gal staining of adipogenic precursors and Oil Red O-positive adipocytes differentiated from Adipoq^+^ lineage cells isolated from control and Cbfβ CKO mice, following vehicle or senolytic (Dasatinib+Quercetin, D+Q) treatment; scale bar, 50 μm. (b) Quantification of β-Gal^+^ cells in adipogenic precursors and mature adipocytes from (a); n=3. (c-d) EdU incorporation in periosteal MSCs treated with conditioned medium (CM) from control or Cbfβ CKO Adipoq^+^ precursors and mature adipocytes, with or without D+Q; quantification in (d); n=4. Scale bar, 50 μm. (e-f) CFU assay of periosteal MSCs treated with CM from control or Cbfβ CKO cells, with or without D+Q; quantification in (f); n=3. Scale bar, 5 mm. (g-j) Western blot analysis of AKT signaling in marrow MSCs treated with CM from adipocyte precursors (g) or mature adipocytes (h), and periosteal MSCs treated with CM from adipocyte precursors (i) or mature adipocytes (j) at the indicated time points after treatment. Data are presented as mean ± SD; p-values determined by two-way ANOVA with Fisher’s LSD multiple comparisons.

**Figure S11 CKO BMACs Suppress Skeletal Stem Cell Proliferation through IGFBP7.**

(a) RT-qPCR detection of *Igfbp7* mRNA in sorted Adipoq^+^ cells from control and Cbfβ CKO mice; n=5. (b-d) Western blot analysis of IGFBP7 protein in sorted Adipoq^+^ cells (b) and in CM from adipogenic precursors (c) and mature adipocytes (d) from control and Cbfβ CKO mice. Bottom panels show total protein in CM by UV imaging. (e-h) EdU incorporation and quantification in bone marrow MSCs (e, f) and periosteal MSCs (g, h) following recombinant IGFBP7 treatment; n=3-4. Scale bar, 50 μm. (i) Western blot analysis of AKT signaling in marrow MSCs stimulated with IGF-1 (100 ng/ml) ± IGFBP7 (100 ng/ml). (j-k) RT-qPCR detection of *Cbfb* and *Igfbp7* expression in Adipoq^+^ cells following lentivirus-mediated sh*Igfbp7* knockdown. (l) Western blot showing IGFBP7 knockdown in Adipoq^+^ cells using sh*Igfbp7*, with protein levels in cells and CM; bottom panel shows total protein in CM by UV imaging. (m-p) Representative EdU incorporation images and quantification of marrow MSCs (m, n) and periosteal MSCs (o, p) treated with CM from shRNA-rescued IGFBP7 Adipoq^+^ cells; n=4. Scale bar, 50 μm. Data are presented as mean ± SD; p-values determined by one-way ANOVA with Fisher’s LSD multiple comparisons.

**Figure S12 *Cbfb* Deletion Severely Reduced Chromatin Accessibility in Adipoq^+^ Cells.**

(a) Quantification of genome-wide ATAC signal intensity, normalized to RPKM. (b) Genome-wide heatmap and average ATAC-seq signal profile centered on TSS. (c) Genomic distribution of ATAC-seq peaks in Cbfβ CKO and control cells. (d) Genomic distribution of Cbfβ binding sites. (e) CUT&Tag-seq analysis of Cbfβ binding sites; genome-wide heatmap and average signal profile centered on peak summits. (f) Genome-wide heatmap and ATAC-seq average signal profile centered on Cbfβ binding loci. (g) Aggregation plot showing ATAC-seq peak numbers around Cbfβ binding loci. (h-i) Venn diagram showing percentage of downregulated genes with reduced chromatin accessibility and upregulated genes with increased chromatin accessibility in CKO Adipoq^+^ cells. (j) IGV tracks of RNA-seq data at the indicated genes. (k) IGV tracks of ChIP-seq signal (GSE79815) at promoters of indicated genes. (l-m) CUT&Tag-qPCR showing reduced RUNX2 occupancy at promoters of *Xrcc5* and *Mad2l2* in CKO Adipoq^+^ cells. Data are presented as mean ± SD; p-values determined by two-tailed unpaired t-test (a) or two-tailed paired *t*-test (l-m).

**Figure S13 Short-Term Cbfβ Deletion Decreases Chromatin Accessibility Independently of Senescence.**

(a) Western blot analysis of Cbfβ protein in marrow MSCs from *Cbfb^f/f^* mice after 8 days of Adenovirus (Ade)-Cre-mediated deletion. (b-c) Representative β-Gal staining (b) and quantification (c) of MSCs following Ade-Cre-induced Cbfβ deletion. n = 4. Scale bar: 50 µm. (d) Genome-wide heatmap and average ATAC-seq signal profile centered on peak summits in MSCs after Ade-Cre-mediated Cbfβ deletion. (e) Quantification of ATAC-seq signal shown in (d). Data are presented as mean ± SD; p-values determined by two-tailed unpaired *t*-test.

**Figure** **S14 Senolytics Rescued Senescence Accumulation, Restored SSPC and Enhanced Bone Healing in *Cbfb^f/f^;Adipoq-Cre* mice.**

(a) Schematic of the femoral fracture mouse model with D+Q treatment and experimental timeline. (b-e) β-Gal staining and quantification of femoral sections (b,c; n=4-6; scale bars: leftmost panels, 200 µm; others 50 µm) and callus section (d,e; n=4-6; scale bars: left panels, 1000 µm; right panels, 100 µm) from control and Cbfβ CKO mice treated with solvent or D+Q. (f-h) Representative IF staining of femoral sections for β-Gal, anti-Adiponectin, and anti-Perilipin‑1 in control and Cbfβ CKO mice treated with solvent or D+Q. Quantification of β-Gal⁺ cells in Adiponectin⁺ Perilipin‑1⁻ (g) and Adiponectin⁺ Perilipin‑1⁺ (h) populations. n=4; scale bar: 10 µm. (i-j) Representative HE staining images and quantification of bone sections. n = 4-7. Scale bar: 50 µm. (k-l) μCT analysis and quantification of callus index. n = 4-10. (m-n) Safranin O/fast green staining and quantification of mineralized callus formation. n = 4-11. Scale bar = 500 µm. (o-q) Flow cytometry analysis and quantification of CD105⁻CD200⁺ SSPCs, and CD105⁺ SSPCs during fracture healing. n =5-8. (r-v) Flow cytometry analysis and quantification of PDGFRα⁺, PDGFRα⁺Sca1⁻, and PDGFRα⁺Sca1⁺ SSPCs during fracture healing. n=5-8. (w) Correlation analysis showing a negative correlation between senescent BMAC proportion and SSPC numbers in the senolytic treatment model. All data are presented as mean ± SD; p-values were determined using one-way ANOVA with Fisher’s LSD multiple comparison in (c, e, g, h, j, l, n, p, q, s, u, v) and Pearson correlation in (w).

**Figure S15 Uncropped blot and gel.**

(a-b, d-n) Uncropped blots were shown. (c) Uncropped gel was shown.

**Materials and Methods**

**Generation of Mice Model**

*Cbfb^f/f^*(B6.129P2-Cbfbtm1Itan/J)*, Adipoq-Cre*(B6.FVB-Tg(Adipoq-cre)1Evdr/J)*,* and *R26^tdTomato^* (B6.Cg-Gt(ROSA)26Sortm9(CAG-tdTomato)Hze/J) mice were obtained from Jackson Laboratory. *Adipoq*-CreERT2 (C57BL/6JGpt-Tg(Adipoq-CreERT2)226/Gpt) mice were obtained from GemPharmatech Co., Ltd. To generate conditional knockout mice, *Cbfb^f/f^* mice, *Adipoq-Cre,* and tdTomato reporter mice were intercrossed, first generating heterozygous offspring, which were subsequently bred to obtain homozygous conditional knockout mice. Littermate *Cbfb^f/f^* mice served as controls for *Cbfb^f/f^;Adipoq-Cre* mice, while *Adipoq-Cre;tdTomato* mice served as controls for *Cbfb^f/f^;Adipoq-Cre;tdTomato* mice. To generate inducible conditional knockout mice, *Cbfb^f/f^* mice were crossed with *Adipoq*-CreERT2 mice and tdTomato reporter mice. The resulting *Cbfb^f/f^;Adipoq-CreERT2;tdTomato* mice were used as inducible knockout mice (iCKO), while littermate *Cbfb^+/+^;Adipoq-CreERT2;tdTomato* mice served as controls. Cre-mediated recombination was induced by intraperitoneal injection of tamoxifen (75 mg/kg body weight, dissolved in corn oil) once daily for three consecutive days, 3 weeks prior to the experiments. Genotyping was performed by PCR using genomic DNA extracted from tail biopsies. All mice were housed in an SPF-level animal facility at Zhejiang University under a 12-hour light/dark cycle with ad libitum access to standard chow and water. Both male and female mice were randomly assigned to experimental groups. All animal procedures were approved by the Institutional Animal Care and Use Committee (IACUC) of Zhejiang University and conducted following the Guide for the Care and Use of Laboratory Animals.

**Generation of the fracture healing model**

As previously described, femoral fractures were induced in 8-week-old mice^1^. Briefly, the femur and patella were surgically exposed, and a 25-gauge syringe needle was inserted parallel to the long axis of the femur through the patellar groove into the marrow cavity. The needle was removed, and a transverse mid-diaphyseal femoral fracture was created using a 0.5-mm Gigli saw. A blunt 25-gauge needle was reinserted through the fracture site into the marrow cavity to stabilize the injury. The injured femur was collected at designated time points for further analysis.

**D+Q senolytic treatment**

For the senolytic treatment, 8-week-old mice were administered a combination of Dasatinib (5 mg/kg, Aladdin, CAS No. 302962-49-8) and Quercetin (50 mg/kg, Aladdin, CAS No. 117-39-5) via weekly oral gavage, following established protocols^2^. The senolytic compounds were prepared in a sterile 1% sodium carboxymethyl cellulose (CMC-Na) solution.

**AAV-mediated Cbfb rescue in middle-aged mice**

To restore *Cbfb* expression in Adipoq⁺ cells during fracture healing, AAV2/9 vectors (pHBAAV-adiponectin-T2A-ZsGreen) driving *Cbfb* under the adiponectin promoter were packaged by Hanbio Biotechnology (Shanghai, China) at a titer of 1.4×10¹² viral genomes (VG)/mL. One month before fracture surgery, young (2-month-old) and middle-aged (9-month-old) mice received 10 μL of AAV via intramedullary injection. Femoral fractures were performed as described above, and callus tissues were harvested at 1 and 3 weeks post-fracture to assess SSPC populations and bone regeneration, respectively.

**ELISA Assays**

Mice were fasted for 8 hours prior to euthanization, and sera were collected for measuring bone turnover markers, collagen type I C-telopeptide degradation products (Sangon Biotech, D721204-0096), and N-terminal propeptide of type I procollagen (Sangon Biotech, D721053-0096) according to the manufacturer's instructions.

**Histology and Tissue Preparation**

At two months of age or following femoral fracture surgery, femurs and tibiae were harvested. Soft tissues were carefully removed, and bones were fixed overnight in 4% paraformaldehyde (PFA; Sbjbio Life Sciences, BL-G002). Samples were then dehydrated and stored in ethanol.

For cryosectioning of undecalcified bones, samples were infiltrated with 30% sucrose in PBS, embedded in a cooled embedding medium (SCEM-L1, Section-Lab) within stainless steel containers, and sectioned at 8 μm using a CryoStar NX50 cryostat (Thermo Fisher Scientific). Sections were mounted on Cryofilm (Section-Lab, Cryofilm type 2C C-FP095) following the Kawamoto method ^3^.

For paraffin and frozen sections, bone samples were decalcified in 10% EDTA (Servicebio, GC202003-500g) for 3 weeks. **Paraffin sections:** Samples were dehydrated in graded ethanol, cleared in xylene, embedded in paraffin, and sectioned at 6 μm using a microtome (KEDEE, KD-3358). Sections were mounted on CITOGLAS adhesive slides (CITOTEST, 188105). **Frozen sections:** Decalcified samples were infiltrated with 30% sucrose in PBS, embedded in optimum cutting temperature (OCT) compound (Thermo Fisher, 6502), and sectioned at 8 μm using a CryoStar NX50 cryostat. Sections were mounted on CITOGLAS adhesive slides (CITOTEST, 188105). Paraffin sections were dried and stored at room temperature, whereas frozen sections were stored at -80°C until further analysis.

**μCT Analysis of Bone and Bone Marrow Adipocyte**

Bone samples were harvested and fixed overnight, as described previously. Micro-computed tomography (μCT) imaging was performed using a BRUKER SKYSCAN 1272 scanner with a 12-μm isotropic voxel size. The scanning region extended from the proximal tibia to the tibiofibular junction. Trabecular bone parameters were analyzed by segmenting the trabecular region (0.4-2.2 mm below the lowest point of the growth plate; 150 slices) using CTan software. The trabecular bone volume fraction (BV/TV) was calculated. For bone healing assessment following femoral fracture, the scan encompassed the entire callus. After image reconstruction, periosteal callus formation at the fracture site was quantified, and fracture gap length was measured. Callus diameter and adjacent diaphyseal diameter were used to calculate the callus index (Callus Diameter / Adjacent Diaphysis Diameter), as previously described ^4,5^.

To evaluate bone marrow adiposity, tibia samples were decalcified in 10% EDTA for 4 weeks, washed with running tap water, and incubated in freshly prepared 1% osmium tetroxide solution for 3 days at room temperature. After incubation, samples were washed with cooled tap water for 2 hours. μCT scanning was performed at a 16-μm isotropic voxel size, covering the entire tibia. Marrow adipose tissue was quantified, and the adipocyte volume per tissue volume (AV/TV) was calculated using CTan software.

All μCT data were analyzed using CTan software. Statistical analysis and data visualization were performed using GraphPad Prism 8. Three-dimensional reconstructions were generated using Amira software.

**Calcein and Alizarin Complexone Labeling**

To assess bone formation dynamics, calcein (10 mg/kg, Sangon Biotech, A602198) and Alizarin Complexone (30 mg/kg, Sangon Biotech, A500010) were administered intraperitoneally to mice at seven and two days before sacrifice, respectively. After euthanasia, tibiae were fixed, embedded without decalcification, and sectioned at 8 μm for frozen sections. Fluorescence images were acquired, and the distance between the two fluorescent labels on the bone surface and the proportion of the bone surface covered by calcein and Alizarin Complexone were measured. The mineral apposition rate (MAR) and bone formation rate (BFR) were calculated as follows: MAR = Distance between Calcein and Alizarin Complexone / interval days; BFR = MAR × (Mineralized Surface / Bone Surface).

**Hematoxylin and Eosin (H&E) Staining**

Paraffin sections were deparaffinized and rehydrated through xylene and a graded ethanol series. Sections were stained with hematoxylin (Sangon Biotech, E607317) for 10 minutes, rinsed in 1% HCl/ethanol for 10 seconds, and counterstained with eosin (Sangon Biotech, E607321) for 2 minutes at room temperature. After staining, sections were dehydrated through a graded ethanol series (95% ethanol and absolute ethanol) and xylene (two changes, 5 seconds each), air-dried, and mounted with neutral balsam (Solarbio, G8590). Brightfield images were acquired using an Olympus VS200 slide scanner. A 0.1 mm² field of view, approximately 0.2 mm below the growth plate, was selected for analysis. The number of bone marrow adipocytes within the selected field was counted, and the adipocyte density (N.Adipocyte/Area) was calculated.

**TRAP Staining**

Paraffin sections were deparaffinized and hydrated through xylene and graded ethanol series. Preparation of TRAP Staining Working Solution: TRAP Working Solution = 6 mg of Fast Red Violet LB Salt (Aladdin, Cas Number: 32348-81-5) + 50 µL of Solution 1 + 10 ml TRAP Incubation Buffer; Solution 1 = 2 mg of Naphthol AS-MX phosphate (Sangon Biotech, A600460-0500) in 100µL Ethylene Glycol Ether. Sections were incubated with TRAP Working Solution at 37℃ 15mins. After staining, the sections were dehydrated and cleared with 95% ethyl alcohol, absolute ethyl alcohol, and xylene (two changes each for 5s), air-dried, and mounted using neutral balsam. The Olympus VS200 slide scanner was used to scan brightfield sections. At approximately 0.2 mm below the growth plate in images, a 0.1 mm^2^ field of view was selected. The number of TRAP-positive osteoclasts, the length of the osteoclast-occupied surface, and the length of the trabecular bone surface were counted and measured. Then, the number of osteoclasts per bone surface(N.Oc/BS) and osteoclast-occupied surface proportion(Oc.S/BS) were calculated.

**Safranin O/fast green Staining**

Frozen sections were thawed at room temperature for 30 minutes, air-dried, and washed with PBS to remove residual OCT embedding medium. Sections were hydrated in distilled water, stained with Weigert’s iron hematoxylin for 10 minutes, and washed under running tap water for 5 minutes. Sections were briefly differentiated in acid ethanol (two dips), washed for 2 minutes, and stained with Fast Green solution for 5 minutes. Excess stain was removed with a quick rinse in 1% acetic acid solution (10 seconds), then staining with Safranin O solution for 10 minutes. After staining, sections were dehydrated through graded ethanol solutions (95% and absolute ethanol) and xylene (two changes, 2 minutes each), air-dried, and mounted with neutral balsam. Brightfield images were acquired using an Olympus VS200 slide scanner. The proportions of Safranin O-positive cartilage callus and mineralized regions in the callus were quantified.

**Senescence-Associated β-Galactosidase (SA-β-Gal) Staining**

Frozen sections were thawed at room temperature for 30 minutes, washed with PBS to remove the OCT embedding medium, and fixed in 4% PFA for 20 minutes at room temperature. The staining solution was prepared according to the kit instructions (Beyotime Senescence β-Galactosidase Staining Kit, C0602; Invitrogen CellEvent Senescence Green Detection Kit, C10851). Sections were incubated with the working solution at 37°C in the dark for 2 hours (Invitrogen kit) or 10 hours (Beyotime kit). Following staining, sections were washed twice with PBS. For brightfield imaging, slides were counterstained with eosin, dehydrated through a graded ethanol series, cleared in xylene, and mounted with neutral balsam (Solarbio, G8590). For fluorescence imaging, slides were counterstained with DAPI (Solarbio, C0065) and mounted with an antifade medium (Beyotime, P0126-25ml). Images were captured using an Olympus VS200 slide scanner. A 0.1 mm² field of view, approximately 0.2 mm below the growth plate or in the bone marrow near the fracture site, was selected for analysis. The number of SA-β-Gal-positive cells, the area of SA-β-Gal-positive regions, and the total area of the field were measured. The senescence cell density (N.β-Gal/Area) and SA-β-Gal-positive area fraction (β-Gal^+^ Area/Total Area) were calculated.

**Immunohistochemistry Staining**

Paraffin sections (6 μm) were deparaffinized, and antigen retrieval was performed by incubating sections in 0.125% trypsin with 0.1% CaCl₂ at 37°C for 20 minutes. Immunohistochemistry staining was conducted using the VECTASTAIN Elite ABC-HRP Kit (Vector Laboratories, PK-6101) and an anti-Osterix antibody (Affinity, DF7731), following the manufacturer’s protocol. Sections were counterstained with hematoxylin, dehydrated through a graded ethanol series, cleared in xylene, and mounted with neutral balsam. Brightfield images were captured using an Olympus VS200 slide scanner. To assess Osterix-positive osteoblasts, a 0.1 mm² field of view, approximately 0.2 mm below the growth plate, was selected. The number of Osterix-positive (Osx⁺) osteoblasts, the length of the Osx⁺ surface, and the total trabecular bone surface length were measured. The number of Osx⁺ cells per trabecular bone surface (N.Osx⁺/BS) and the proportion of Osx⁺ surface relative to total trabecular bone surface (Osx⁺.BS/BS) were calculated.

**Immunofluorescence (IF) Staining**

Frozen sections were thawed at room temperature for 30 minutes and washed with PBS to remove the OCT embedding medium. Sections were then fixed in 4% paraformaldehyde (PFA) for 20 minutes at room temperature, followed by antigen retrieval in 0.125% trypsin with 0.1% CaCl₂ at 37°C for 20 minutes. Slides were blocked with 5% goat serum (Beyotime, C0265) in universal block buffer (UBB: 0.3% Triton X-100, 1% BSA, and 0.05% NaN₃ in PBS) for 1 hour at room temperature. Immunofluorescence staining was performed using the following primary antibodies: anti-p21 (Proteintech, 82669-2-RR), anti-Cbfβ (CST, 62184S), anti-Perilipin-1 (CST, 9349), anti-OPN (Beyotime, AF7665), anti-PDGFRα (Invitrogen, 13-1401-82), anti-F4/80 (BioLegend, 123105), anti-MPO (R&D Systems, BAF3667), anti-Adiponectin (Proteintech, 66239-1-Ig), and CD31(BD Pharmingen, 557355). For p21, Cbfβ, Perilipin-1 and OPN, sections were incubated with Alexa Fluor 647-conjugated secondary antibody (Invitrogen, A-21245). For CD31, sections were incubated with Alexa Fluor 647-conjugated (CST,4418) or Cy3-conjugated (Yeasen, 33308ES60) secondary antibody. For PDGFRα, F4/80, and MPO, endogenous biotin was blocked (Beyotime, P0101), and Alexa Fluor 647-conjugated streptavidin (Invitrogen, S21374) was used. For adiponectin, endogenous mouse IgG was blocked using the MOM kit (Vector Laboratories, BMK2202), followed by Alexa Fluor 594-conjugated secondary antibody (Invitrogen, A-11005). Sections were incubated with primary antibodies overnight at 4°C, washed with PBST (0.5% Tween-20), and then incubated with the appropriate secondary antibodies for 1 hour at room temperature, protected from light. Slides were counterstained with DAPI, mounted with antifade medium, imaged using the Olympus VS200 slide scanner and FV3000 confocal microscope, and analyzed with OlyVia software. Positive signals were assessed by comparison with negative control sections lacking the primary antibody.

**Flow Cytometry Analysis of SSPCs**

Flow cytometry was performed using a NovoCyte Advanteon flow cytometer (Agilent, Advanteon V6B5R3), and data were analyzed with NovoExpress software. Flow cytometry analysis followed the protocol established by Charles K.F. Chan ^4,6-8^.

Briefly, femurs or bone calluses were dissected, cleaned of soft tissue, and dissociated using a combination of mechanical and enzymatic methods. The tissue was finely minced in a mortar and incubated in collagenase digestion buffer (1 mg/ml collagenase type I [Gibco, 17018029], 1 mg/ml Dispase II [Sigma, D4693-1g], 1 mg/ml BSA, 1% HEPES [Beyotime, C0215-100ml], 5 mM EDTA [Invitrogen, am9269G], and 1% Antibiotic-Antimycotic [Gibco, 15240062] in αMEM) at 37°C for 30 minutes with constant agitation. For periosteal SSPC analysis, the periosteum was peeled from the long bone and was digested. Following digestion and neutralization, cells were collected by gentle pipetting, and the supernatant was transferred to a new tube. The remaining undigested tissue fragments were resuspended in a fresh digestion buffer and subjected to a second round of digestion. The total dissociated cells were filtered through a 70-µm nylon mesh and pelleted by centrifugation at 200 × g at 4°C for 5 minutes. Red blood cells were lysed with ACK lysing buffer (Beyotime, C3702-500ml), and the remaining cells were centrifuged again. The pellet was resuspended in 100 µl FACS buffer and stained with fluorescent-conjugated antibodies for 60 minutes at 4°C. Antibody details are provided in the 'Key Resources' table. Live cells were identified based on DAPI exclusion (1:1000 dilution from a 1 mg/ml stock). Compensation and fluorescence-minus-one (FMO) control-based gating were performed before data acquisition using specific antibody combinations, as indicated in the respective figure legends.

**Isolation of Mouse Bone Marrow Adipose Tissue-Enriched Fraction**

Tibiae were harvested and cut at the fibula junction. The distal tibial bone marrow was extracted by centrifugation at 4°C and 7000 rpm. The bone marrow was resuspended in PBS and filtered through a 70-µm cell strainer. The bone marrow adipose tissue-enriched fraction remained on the filter while blood and stromal cells passed through. This procedure was repeated 2-3 times until no visible red marrow cells remained on the filter. The bone marrow adipose tissue-enriched fraction was lysed using RNAiso Plus (Takara, 9109) for subsequent analysis.

**Isolation and Culture of Bone Marrow Mesenchymal Stem Cells (MSCs)**

After euthanasia, tibiae and femurs were harvested and washed three times with PBS containing 1% Antibiotic-Antimycotic (Gibco, 15240062). Bone marrow was exposed by cutting the bone ends, and marrow cells were collected by placing the bones in a 0.6-ml centrifuge tube with a small hole at the bottom, nested within a 1.5-ml tube, followed by centrifugation at 7000 rpm for 10 seconds. The periosteum was peeled from the long bone and was subjected to collagenase digestion (2 mg/ml collagenase type I [Gibco, 17018029], 0.25% trypsin [Gibco, 15090046], dissolved in αMEM with HEPES and Antibiotic-Antimycotic) at 37°C for 30 minutes. Digestion was halted by adding a complete growth medium containing fetal bovine serum (FBS). Cells were gently resuspended, and the process was repeated with PBS to collect additional cells. Isolated bone marrow and periosteal cells were plated separately in 10-cm culture dishes and maintained in growth medium. These primary cells were designated as passage 0 (P0). The culture medium was changed every 2-3 days. After 7 days, when cell colonies reached confluence, cells were detached with 0.25% Trypsin-EDTA (Gibco, 25200072) for 3 minutes at room temperature, centrifuged at 1000 rpm for 5 minutes, and reseeded in new 10-cm dishes. MSCs were enriched after two passages (P2) and used for adipogenesis and other experiments.

**Adipogenesis and Conditioned Medium (CM) Collection**

Adipoq⁺ precursors were differentiated in adipogenic medium supplemented with 1 μM Rosiglitazone (Sigma, CAS No. 122320-73-4), 1 μM Dexamethasone (Sigma, CAS No. 50-02-2), 500 μM IBMX (Beyotime, SC0195-10mM), and 10 μg/mL Insulin (Beyotime, P3378-20mg) for 3-4 days until mature lipid-laden adipocytes form. Before or following differentiation, the adipogenic medium was removed, and cells were cultured in fresh αMEM for 48 hours. Conditioned medium (CM) was then collected for further analysis. For SASP suppression, cells were treated with Dasatinib (200 nM) and Quercetin (50 μM) for 24 h before being replaced with fresh medium for CM collection. To rescue IGFBP7 levels in CM, cells were infected with lentiviral shRNA targeting *Igfbp7* (sh*Igfbp7*) 4 days prior to CM collection.

**Adenovirus (Ade)-mediated Deletion of *Cbfb* in *Cbfb^f/f^* Bone Marrow MSCs**

Bone marrow MSCs were isolated from *Cbfb^f/f^* mice and expanded to passage 2 (P2). At 80% confluence, Cells were infected with Ade-Cre (2.2 × 10¹⁰ PFU/mL) at a multiplicity of infection (MOI) of 44. After 24 h, the medium was replaced, and adipogenic differentiation was initiated. Cells were collected 8 days post-infection for downstream analyses.

**Isolation of Human Marrow Adipose Tissue(hMAT) and Human Mesenchymal Stem Cells(hMSCs)**

Bone marrow aspirates (5 mL) were diluted with 45 mL PBS containing 1% Antibiotic-Antimycotic (Gibco, 15240062) and centrifuged at ~600g for 10 min. The floating hMAT and the cell pellet were collected separately and washed once with antibiotic-containing PBS, followed by centrifugation at ~600g for 10 min. The hMAT fraction was fixed in 4% paraformaldehyde (PFA) for 12 h at 4 ℃, washed with PBS, and pre-embedded in agarose to facilitate handling of the small adipose fragments. Samples were subsequently processed through graded dehydration and paraffin embedding, and 6-μm paraffin sections were prepared for histological staining. The cell pellet was resuspended and cultured in α-MEM complete medium supplemented with 10% FBS and 1% Antibiotic-Antimycotic. The medium was replaced every 2-3 days. Human mesenchymal stem cells (hMSCs) were enriched and purified through two passages using 0.25% Trypsin-EDTA (Gibco, 25200072). Passage 2 (P2) cells were used for adipogenic differentiation assays. This study was approved by the Ethics Committee of the Second Affiliated Hospital, Zhejiang University School of Medicine (Ethics approval No.: (2022)伦审研第(0524)号; Project No.: 2022-0216; Application No.: IR2022181).

**Colony-Forming Unit (CFU) Assay**

P0 cells were seeded at a density of 1 × 10⁶ cells per well in 6-well plates and cultured for 8-9 days in growth, adipogenic, or osteogenic medium. Cells were then fixed in 4% paraformaldehyde (PFA) for 20 minutes at room temperature and stained with crystal violet, Oil Red O, or ALP staining to assess total colony-forming units (CFU), MSC subpopulations with adipogenic potential (AD-CFU), and MSC subpopulations with osteogenic potential (OB-CFU), respectively. Brightfield images were captured using the EVOS FLAuto2 imaging system (Thermo Fisher) and stitched together for analysis. Colonies containing at least 20 cells were counted, and the number of CFU, AD-CFU, and OB-CFU colonies was quantified. Total CFUs were visualized by crystal violet staining. OB-CFU and AD-CFU were identified by alkaline phosphatase (ALP) staining and Oil Red O (ORO) staining, respectively. AD-CFUs were defined as colonies in which more than 50% of the cells were ORO-positive.

**Crystal Violet Staining**

Cells were fixed in 4% PFA for 20 minutes at room temperature, stained with 0.5% Crystal Violet solution (MeilunBio, MA0148) for 20 minutes, and washed three times with PBS.

**Oil Red O Staining**

Cells were fixed in 4% PFA for 20 minutes, stained with Oil Red O working solution (0.05% Oil Red O in 40% isopropanol; Aladdin, CAS No. 1320-06-5) for 20 minutes, and washed three times with PBS.

**ALP Staining**

Cells were fixed in 4% PFA for 20 minutes, stained using the BCIP/NBT Alkaline Phosphatase Color Development Kit (MeilunBio, MA0197) for 5 minutes, and washed three times with PBS.

**Flow Cytometry Sorting of Adipoq+ cells**

Tomato^+^ cells were isolated from *Adipoq-Cre;tdTomato* mouse bone marrow using Flow cytometry sorting was performed using a FACS Aria II cell sorter (BD Biosciences), collected in growth medium, and seeded at a density of 60,000 cells per well in a 24-well plate for further analysis.

**EdU Incorporation Assay**

The EdU incorporation assay was performed using the BeyoClick™ EdU Cell Proliferation Kit with Alexa Fluor 488 (Beyotime, C0071S) and the Click-iT™ Plus EdU Alexa Fluor 488 Flow Cytometry Assay Kit (Invitrogen, C10632).

For in vitro MSC EdU incorporation, MSCs (50,000 cells per well) were seeded in 24-well plates with chamber slides. After cell attachment, cells were cultured in conditioned medium (CM) for 48 hours, followed by incubation with 10 µM EdU. The cells were then fixed, and the EdU click reaction was performed according to the manufacturer’s instructions (Beyotime). Finally, cells were counterstained with DAPI (Solarbio, C0065), mounted with an antifade medium, and imaged using an Olympus FV3000 confocal microscope. The number of EdU-positive cells was quantified.

For in vivo proliferation analysis, EdU (50 mg/kg) was intraperitoneally injected into mice 20 hours before sacrifice. Following surface marker staining of skeletal stem and progenitor cells (SSPCs), EdU staining was performed according to the manufacturer’s protocol (Thermo Fisher), and flow cytometry analysis was conducted.

**Telomere-associated Foci (TAF) Analysis**

To assess DNA damage in vitro, tdTomato⁺ cells were isolated via sorting from *Adipoq-Cre;tdTomato* and *Cbfb^f/f^Adipoq-Cre;tdTomato* mice and cultured. A total of 60,000 cells per well were seeded in 24-well plates with chamber slides. After attachment, cells were treated with 100 nM H₂O₂ for 8 hours to induce DNA damage, followed by 40 hours of recovery in growth medium. TAF detection was performed via fluorescence in situ hybridization (FISH) using TelC-Alexa-488 (Panagene, F1004) and immunofluorescence (IF) with an anti-γH2A.X antibody (Abclonal, AP0687). Following the manufacturer’s protocol, cells were subjected to sequential hybridization and immunostaining procedures. Imaging was performed using an Olympus FV3000 confocal microscope, and TAF-positive cells were quantified. The mean number of TAFs per cell was analyzed using FIJI (ImageJ; NIH, Bethesda, MD, USA). Cells containing ≥3 TAFs were classified as DNA damage based on established criteria^2,9,10^.

**Western Blot Analysis**

Protein was extracted from Adipoq⁺ cells using RIPA lysis buffer (Beyotime, P0013B) supplemented with a protease and phosphatase inhibitor cocktail (Beyotime, P1045) and 5×SDS-PAGE protein loading buffer (Yeason, 20315ES05). SDS-PAGE was performed, and proteins were transferred onto PVDF membranes. Protein levels of Cbfβ, p21, perilipin-1, γ-H2A.X, IGFBP7 and Adiponectin were analyzed using the following primary antibodies: rabbit anti-Cbfβ (Cell Signaling Technology, 62184S), rabbit anti-p21 (Proteintech, 82669-2-RR), rabbit anti-perilipin-1 (Proteintech, 83905-4-RR), rabbit anti-γ-H2A.X (Abclonal, AP0687), rabbit anti IGFBP7(proteintech, 19961-1-AP), and mouse anti-Adiponectin (Proteintech, 66239-1-Ig). HRP-conjugated anti-rabbit IgG (FUDE Biological Technology, FDR007) was used as the secondary antibody, and chemiluminescence detection was performed. Protein concentrations of conditioned media were quantified using a BCA Protein Assay Kit (Beyotime, P0012). Equal amounts of protein (20 μg per lane) were subjected to SDS-PAGE. Total protein loading was visualized using stain-free gels (EpiZyme, PG223) and imaged under UV illumination.

**RNA Extraction, Reverse Transcription, and qRT-PCR Analysis**

Total RNA was extracted from cells and tissues using RNAiso Plus (Takara, 9109) according to the manufacturer’s instructions. cDNA synthesis was performed using 4× Hifair III SuperMix Plus (Yeasen, 11141ES60) with 1 μg of total RNA. qRT-PCR was conducted using Hieff qPCR SYBR Green Master Mix (Yeasen, 11203ES08). Primer sequences are listed in Table S1.

**RNA-seq and ATAC-seq**

RNA sequencing (RNA-seq) and assay for transposase-accessible chromatin using sequencing (ATAC-seq) were performed on sorted and cultured Adipoq⁺ cells isolated from *Adipoq-Cre;tdTomato* and *Cbfb^f/f^;Adipoq-Cre;tdTomato* mice. The experiments and data analysis were conducted by SEQHEALTH Biotech Company (Wuhan, China).

For RNA-seq, total RNA was extracted using RNAiso Plus (Takara, 9109) according to the manufacturer’s protocol. RNA quality was assessed by measuring the A260/A280 ratio, with RNA integrity evaluated based on the RNA Integrity Number (RIN). Only RNA samples with A260/A280 > 1.6 and RIN > 7 were used for sequencing. Differentially expressed genes (DEGs) were identified using statistical thresholds of q < 0.05 and |log₂(FC)| > 1. Further analyses included Gene Set Enrichment Analysis (GSEA) performed using GSEA 4.0.3 software, as well as Gene Ontology (GO) enrichment analysis conducted with the clusterProfiler R package using the org.Mm.eg.db annotation database for Mus musculus^11^. Data visualization was performed using the LC-Bio Cloud Platform (https://www.omicstudio.cn/).

For ATAC-seq, cell samples were lysed using a lysis buffer provided by SEQHEALTH Biotech Company, and nuclei were isolated for transposition reactions. DNA fragments generated from the transposition reaction were used for library preparation and high-throughput sequencing. ATAC-seq data were aligned to the GRCm39 (Mus musculus) reference genome. ATAC-seq tag intensity matrices were computed in a 50 bp bin size, and signal visualization over the whole genome, transcription start sites (TSS), and Cbfβ target loci was performed using deepTools^12^. Peak calling was performed using MACS2 with the call-summits method and default parameters (p-value cutoff of 1e-5)^13^. Genomic distances between ATAC-seq peaks and their nearest Cbfβ CUT&Tag peak summits were computed using the GenomicRanges package^14^. Subsequently, the spatial distribution of ATAC-seq peak densities surrounding Cbfβ target loci was quantitatively visualized using the ggplot2 package^15^. Genomic distribution and motif enrichment analyses were conducted using AME (Analysis of Motif Enrichment) tool, and motif scanning within defined genomic regions was performed using the FIMO (Find Individual Motif Occurrences) tool from the MEME Suite (<https://meme-suite.org/meme/tools/fimo>). The raw sequencing data generated in this study have been deposited in the Gene Expression Omnibus (GEO) database under the accession number GSE293612.

**CUT&Tag-seq**

Cleavage under targets and tagmentation sequencing (CUT&Tag-seq) was performed on primary mesenchymal stem cells (MSCs) isolated from 8-week-old wild-type mice. MSCs were transduced with lentivirus carrying the pLenti-Cbfb overexpression plasmid to achieve Cbfβ overexpression. The pLenti-Cbfb plasmid was constructed by cloning the Cbfβ coding sequence into the pLenti-puro vector (Addgene, Plasmid #17452), with the empty vector serving as a control.

CUT&Tag was conducted using the Hyperactive Universal CUT&Tag Assay Kit for Illumina Pro (Vazyme, Cat# TD904) following the manufacturer’s protocol. Briefly, cells were immobilized on Concanavalin A beads, permeabilized, and incubated with anti-Cbfβ antibody (Cell Signaling Technology, 62184S), and treated with hyperactive Tn5 transposase preloaded with adapters to fragment DNA at protein-binding sites. The resulting libraries were purified, PCR-amplified, and sequenced on the Illumina platform for high-resolution profiling of protein-DNA interactions. Sequencing was performed on the Illumina NovaseqTM 6000 platform by LC-Bio Technologies (Hangzhou) Co., Ltd., and raw data were processed using the Vazyme Cloud Platform (<http://cloud.vazyme.com:83/>). Data alignment was conducted using the GRCm39 (Mus musculus) genome, and peak visualization was performed using the Integrative Genomics Viewer (IGV). The raw sequencing data generated in this study have been deposited in the Gene Expression Omnibus (GEO) database under the accession number GSE293612.

**Chromatin Immunoprecipitation (ChIP)**

ChIP was performed on MSCs overexpressing Cbfβ after adipogenic differentiation. Cells were crosslinked with 1% formaldehyde (Macklin, CAS No. 50-00-0) at room temperature for 15 minutes, followed by quenching with 125 mM glycine. Cells were collected and subjected to nuclear fractionation by grinding in subcellular fractionation buffer (20 mM HEPES, 10 mM KCl, 2 mM MgCl₂, 1 mM EDTA, and 1 mM EGTA in ddH₂O) supplemented with a protease and phosphatase inhibitor cocktail and 1 mM DTT (Beyotime, ST043). Nuclei were lysed in ChIP lysis buffer (50 mM Tris-HCl, pH 8.0, 1% SDS, 10 mM EDTA) on ice for 20 minutes. Chromatin was fragmented to 200-1000 bp via sonication. Chromatin immunoprecipitation was performed using an anti-Cbfβ antibody. Immunoprecipitated DNA was purified using the FastPure Gel DNA Extraction Mini Kit (Vazyme, DC301-01). Quantitative PCR (qPCR) was performed on immunoprecipitated DNA to assess the binding of Cbfβ to target gene promoters. Primers targeting the *Xrcc5* and *Mad2l2* promoter regions were used (primer sequences are listed in Table S2).

**Statistical Analysis**

Data are presented as mean ± standard deviation (SD). Statistical significance was determined using a Two-tailed Student’s t-test for comparisons between two groups, One-way ANOVA followed by Fisher’s LSD post hoc test for single-factor multiple-group comparisons, or Two-way ANOVA with Fisher’s LSD post hoc test for two-factor comparisons (e.g., genotype and sex) as indicated in the figure legends. All statistical analyses were performed using GraphPad Prism 8, with a significance threshold of p < 0.05. P-values are indicated in the figure between bars. Sample numbers (N) of statistical data and representative data are all indicated in the figure legends.

**Data availability**

All data supporting the findings of this study are available within the article and its supplementary information. Raw sequencing data have been deposited in the NCBI Gene Expression Omnibus under accession number GSE293612.

| **Key Resources Table** | | | | |
| --- | --- | --- | --- | --- |
| **Reagent type**（species）or resource | **Designation** | **Source or reference** | **Identifiers** | **Additional information** |
| Strain | B6.129P2-*Cbfb*^tm1Itan^/J | The Jackson Laboratory | Strain #:008765 | RRID: IMSR_JAX:008765 |
| Strain | B6.Cg-*Gt(ROSA)26Sor^tm9^(CAG-tdTomato)Hze*/J | The Jackson Laboratory | Strain #:007909 | RRID: IMSR_JAX:007909 |
| Strain | B6.FVB-Tg(Adipoq-cre)1Evdr/J | The Jackson Laboratory | Strain #:028020 | RRID: IMSR_JAX:028020 |
| Strain | C57BL/6JGpt-Tg(Adipoq-CreERT2)226/Gpt | GemPharmatech Co., Ltd. | Strain NO. T052679 | MGI:106675 |
| Antibody | Anti-γH2A.X-antibody | Abclonal | AP0687 | IF: 1:100  WB: 1:1000 |
| Antibody | Anti-Osterix antibody | Affinity | DF7731 | IHC: 1:100 |
| Antibody | BD OptiBuild^TM^ BV650 Rat Anti-Mouse CD200 | BD Biosciences | 745402 | FACS: 1:75 |
| Antibody | PerCP/Cyanine5.5 anti-mouse CD31 antibody | Biolegend | 102420 | FACS: 1:300 |
| Antibody | PerCP/Cyanine5.5 anti-mouse CD45 antibody | Biolegend | 102132 | FACS: 1:300 |
| Antibody | PerCP/Cyanine5.5 anti-mouse TER-119/erythroid cells antibody | Biolegend | 116228 | FACS: 1:500 |
| Antibody | Alexa Fluor 647 anti-mouse Ly51 Antibody | Biolegend | 108311 | FACS: 1:200 |
| Antibody | PE/Cyanine7 anti-mouse CD105 Antibody | Biolegend | 120409 | FACS: 1:100 |
| Antibody | Anti-Cbfβ-antibody | Cell Signaling Technology | 62184S | WB: 1:2000; IF: 1:150; ChIP/CUT&Tag: 1:100 |
| Antibody | CD90.2(Thy-1.2) Monoclonal Antibody(53-2.1), APC-eFlour^TM^ 780 | eBioscience^TM^ | 47-0902-82 | FACS: 1:500 |
| Antibody | Ly-6A/E (Sca-1) Monoclonal Antibody (D7), FITC | eBioscience^TM^ | 11-5981-82 | FACS: 1:100 |
| Antibody | CD140a (PDGFRA) Monoclonal Antibody (APA5), APC | eBioscience^TM^ | 17-1401-81 | FACS: 1:75 |
| Antibody | Alexa Fluor 647 conjugated secondary antibody | Invitrogen | A-21245 | IF: 1:400 |
| Antibody | Alexa Fluor 594 conjugated secondary antibody | Invitrogen | A‑11005 | IF: 1:400 |
| Antibody | Alexa Fluor 647 conjugated Streptavidin | Invitrogen | S21374 | IF: 1:400 |
| Antibody | Cy3 conjugated secondary antibody | Yeasen | 33308ES60 | IF: 1:400 |
| Antibody | Alexa Fluor 647 conjugated secondary antibody | CST | 4418 | IF: 1:400 |
| Antibody | Anti-p21-antibody | Proteintech | 82669-2-RR | WB: 1:2000; IF: 1:150 |
| Antibody | Anti-perilipin-1-antibody | Proteintech | 83905-4-RR | WB: 1:2000 |
| Antibody | Anti-Perilipin-1-antibody | CST | 9349 | IF: 1:200 |
| Antibody | Anti-PDGFRα-antibody | Invitrogen | 13-1401-82 | IF: 1:100 |
| Antibody | Anti-F4/80-antibody | Biolegend | 123105 | IF: 1:100 |
| Antibody | Anti-MPO-antibody | R&D Systems | BAF3667 | IF: 1:200 |
| Antibody | Anti-Adiponectin-antibody | Proteintech | 66239-1-Ig | IF: 1:200 |
| Antibody | Anti-IGFBP7-antibody | Proteintech | 19961-1-AP | WB: 1:1000 |
| Antibody | Anti-OPN-antibody | Beyotime | AF7665 | IF: 1:100 |
| Antibody | Anti-CD31-antibody | BD Pharmingen | 557355 | IF: 1:100 |
| Cytokines | IGFBP-7 | MCE | HY-P70858 | 100ng/ml |
| Cytokines | IGF-I Protein | Abclonal | RP00996 | 100ng/ml |
| Commercial assay or kit | BCA Protein Assay Kit | Beyotime | P0012 |  |
| Commercial assay or kit | Omni-Easy™ One-Step Fast PAGE Gel Preparation Kit (Stain-Free) | Epizyme |  |  |
| Commercial assay or kit | M.O.M. (Mouse on Mouse) Immunodetection Kit, Basic | Vector Laboratories | BMK2202 |  |
| Commercial assay or kit | VECTASTAIN Elite ABC-HRP Kit, Peroxidase, Rabbit IgG | Vector Laboratories | PK-6101 |  |
| Chemical compound | Oil Red O | Aladdin | Cas Number:1320-06-5 |  |
| Chemical compound | Dasatinib | Aladdin | Cas Number: 302962-49-8 |  |
| Chemical compound | Quercetin | Aladdin | Cas Number: 117-39-5 |  |
| Chemical compound | Antifade mounting medium | Beyotime | P0126-25ml |  |
| Chemical compound | HEPES | Beyotime | C0215-100ml |  |
| Chemical compound | IBMX | Beyotime | SC0195-10mM |  |
| Chemical compound | Insulin | Beyotime | P3378-20mg |  |
| Chemical compound | Red Blood Cell Lysis Buffer | Beyotime | C3702-500ml |  |
| Chemical compound | Goat serum | Beyotime | C0265 |  |
| Chemical compound | RIPA lysis buffer | Beyotime | P0013B |  |
| Chemical compound | Protease and phosphatase inhibitor cocktail | Beyotime | P1045 |  |
| Chemical compound | SDS | Beyotime | ST626 |  |
| Chemical compound | Tris-HCL ph8 | Beyotime | ST780 |  |
| Chemical compound | EGTA | Beyotime | ST068 |  |
| Chemical compound | CITOGLAS adhesive slides | CITOTEST | 188105 |  |
| Chemical compound | Fixable Viability Dye eFluor™ 780 | eBioscience™ | 65-0865-14 |  |
| Chemical compound | Antibiotic-Antimycotic | Gibco | 15240062 |  |
| Chemical compound | Trypsin-EDTA (0.25%) solution | Gibco | 25200072 |  |
| Chemical compound | Collagenase type I | Gibco | 17018029 |  |
| Chemical compound | Trypsin | Gibco | 15090046 |  |
| Chemical compound | αMEM | Gibco | 11900024 |  |
| Chemical compound | GlutaMAX | Gibco | REF35050-061 |  |
| Chemical compound | BODIPY 493/503 | Invitrogen | D3922 |  |
| Chemical compound | EDTA | Invitrogen | AM9269G |  |
| Chemical compound | 3.7% Formaldehyde in H2O | Macklin | CAS Number: 50-00-0 |  |
| Chemical compound | Crystal Violet Staining Solution | MeilunBio | MA0148 |  |
| Chemical compound | Calcein | Sangon Biotech | A602198 |  |
| Chemical compound | Alizarin complexone | Sangon Biotech | A500010 |  |
| Chemical compound | Hematoxylin | Sangon Biotech | E607317 |  |
| Chemical compound | Eosin | Sangon Biotech | E607321 |  |
| Chemical compound | TritonX-100 | Sangon Biotech | A110694-0100 |  |
| Chemical compound | 4% Paraformaldehyde | Sbjbio life Sciences | BL-G002 |  |
| Chemical compound | SCEM-L1 | Section-Lab | [section-lab.jp/Japanese/Technic/Material.htm](http://section-lab.jp/Japanese/Technic/Material.htm) |  |
| Chemical compound | Cryofilm type 2C | Section-Lab | C-FP095 |  |
| Chemical compound | Edetate Disodium | Servicebio | GC202003-500g |  |
| Chemical compound | β-glycerophosphate | Sigma | Cas Number:154804-51-0 |  |
| Chemical compound | L-ascorbic acid | Sigma | Cas Number:50-81-7 |  |
| Chemical compound | Rosiglitazone | Sigma | Cas Number:122320-73-4 |  |
| Chemical compound | Dexamethasone | Sigma | Cas Number:50-02-2 |  |
| Chemical compound | Dispase II | Sigma | D4693-1g |  |
| Chemical compound | Glucose | Sigma | Cas Number: 50-99-7 |  |
| Chemical compound | Neutral balsam | Solarbio | G8590 |  |
| Chemical compound | DAPI | Solarbio | C0065 |  |
| Chemical compound | RNAiso Plus | Takara | 9109 |  |
| Chemical compound | OCT | Thermo Fisher Scientific | 6502 |  |
| Commercial assay or kit | Hyperactive Universal CUT&Tag Assay Kit for Illumina Pro (Vazyme, Cat# TD904) | Vazyme | Cat# TD904 |  |
| Commercial assay or kit | Hifair Ⅲ SuperMix plus | Yeasen | 11141ES60 |  |
| Commercial assay or kit | Hieff qPCR SYBR Green Master Mix | Yeasen | 11203ES08 |  |
| Commercial assay or kit | Senescence β-Galactosidase Staining Kit | Beyotime | C0602 |  |
| Commercial assay or kit | BeyoClick™ EdU Cell Proliferation Kit with Alexa Fluor 488 | Beyotime | C0071S |  |
| Commercial assay or kit | CellEvent Senescence Green Detection Kit | Invitrogen | C10851 |  |
| Commercial assay or kit | Click-iT™ Plus EdU Alexa Fluor™ 488 Flow Cytometry Assay Kit ­­­ | Invitrogen | C10632 |  |
| Commercial assay or kit | Alkaline Phosphatase Color Development Kit | MeilunBio | MA0197 |  |
| Commercial assay or kit | Toluidine Blue Staining Kit | Sangon Biotech | NO. E670105 |  |
| Commercial assay or kit | Collagen type I C-telopeptide degradation products | Sangon Biotech | D721204-0096 |  |
| Commercial assay or kit | N-terminal propeptide of type I procollagen products | Sangon Biotech | D721053-0096 |  |
| Commercial assay or kit | FastPure Gel DNA Extraction Mini Kit | Vazyme | DC301-01 |  |
| Instrument | NovoCyte Advanteon flow cytometer | Agilent | Adcanteon V6B5R3 |  |
| Instrument | FACS Aria II cell sorter | BD Biosciences | BD FACS ARIA II |  |
| Instrument | SKYSCAN | BRUKER | 1272 |  |
| Instrument | CryoStar NX50 | Thermo Fisher Scientific | CryoStar NX50 |  |
| Sequence-based reagent | TelC-Alexa-488 | Panagene | F1004 |  |
| Instrument | Microtome | KEDEE | KD3358 |  |
| Chemical compound | 5×SDS-PAGE protein loading buffer | Yeason | 20315ES05 |  |
| Plasmid | pLenti CMV Puro DEST | Addgene | #17452 |  |
| Instrument | VS200 | Olympus |  |  |
| Instrument | FV3000 | Olympus |  |  |
| Software | CTan | BRUKER |  |  |
| Software | Amira | Thermo Fisher Scientific |  |  |
| Software | Graphpad prism 8 |  |  |  |
| Software | Image J |  |  |  |
| Software | Novo Express | Agilent |  |  |
| Software | OlyVIA | Olympus |  |  |

| **Table S1 Primers used for qPCR** | | |
| --- | --- | --- |
| **Gene** | **Forward primers** | **Reverse primers** |
| *Actb* | GGCTGTATTCCCCTCCATCG | CCAGTTGGTAACAATGCCATGT |
| *Cbfb* | GGCCTTTGAAGAGGCTCGAA | TGCTGTCTTCTTGCCAGTTAC |
| *Ccl2* | TTAAAAACCTGGATCGGAACCAA | GCATTAGCTTCAGATTTACGGGT |
| *Ccl3* | TCCCAGCCAGGTGTCATTTT | TTGGAGTCAGCGCAGATCTG |
| *Ccl7* | CCCTGGGAAGCTGTTATCTTCA | CTGATGGGCTTCAGCACAGA |
| *Ccl8* | TCTACGCAGTGCTTCTTTGCC | AAGGGGGATCTTCAGCTTTAGTA |
| *Ccnd1* | GCGTACCCTGACACCAATCTC | CTCCTCTTCGCACTTCTGCTC |
| *Cdk18* | TTGAGGAGTCGTTGGCTGAG | GGTTCTGCCGTTGTTGATACT |
| *Cdkn1a* | CCTGGTGATGTCCGACCTG | CCATGAGCGCATCGCAATC |
| *Cxcl12* | GCCAACGTCAAGCATCTGAAA | CAGCCGTGCAACAATCTGAA |
| *Cxcl13* | GGCCACGGTATTCTGGAAGC | GGGCGTAACTTGAATCCGATCTA |
| *Cxcl5* | TCCAGCTCGCCATTCATGC | TTGCGGCTATGACTGAGGAAG |
| *Fabp4* | AGCACCATAACCTTAGATGGGG | CGTGGAAGTGACGCCTTTCA |
| *Hprt1* | GGTGGAGATGATCTCTCAACTTTAA | GGGAAAGCAAAGTTTGCATTGTT |
| *Igfbp3* | CCAGGAAACATCAGTGAGTCC | GGATGGAACTTGGAATCGGTCA |
| *Il1b* | TCAGGCAGGCAGTATCACTCA | CACGGGAAAGACACAGGTAGCT |
| *Il4* | TCCTCACAGCAACGAAGAACAC | AAGCACCTTGGAAGCCCTACA |
| *Il7* | TTCCTCCACTGATCCTTGTTCT | AGCAGCTTCCTTTGTATCATCAC |
| *Igfbp7* | AAGAGGCGGAAGGGTAAAGC | TGGGGTAGGTGATGCCGTT |
| *Rbp4* | AGTCAAGGAGAACTTCGACAAGG | CAGAAAACTCAGCGATGATGTTG |
| *Mad2l2* | CTCTATGTGCGCGAGGTCTAC | CACTGGGCGGTGTTCCTTAT |
| *Mki67* | ATCATTGACCGCTCCTTTAGGT | GCTCGCCTTGATGGTTCCT |
| *Mmp9* | TGAGTCCGGCAGACAATCCT | CCCTGGATCTCAGCAATAGCA |
| *Smc5* | TTCCAGACACAAGTACCCACC | TCCTCAACCACCGAAGGATTC |
| *Timp2* | TCAGAGCCAAAGCAGTGAGC | GCCGTGTAGATAAACTCGATGTC |
| *Tnf* | CCTGTAGCCCACGTCGTAG | GGGAGTAGACAAGGTACAACCC |
| *Xrcc5* | CAGACACCTGATGCTACCAGA | GCTGAATCAAATCCATGCACACA |

| **Table S2 Primers used for ChIP assay** | | |
| --- | --- | --- |
| **Gene** | **Forward primers** | **Reverse primers** |
| *Xrcc5* p1 | CATGGCGTGGTCCGGTAATA | TCCGAACCCCAAGTCTTTCG |
| *Xrcc5* p2 | TCGGTGAACTCTCACTCTTCTC | TCTGGAGTTAAAAGTCCCCTGTG |
| *Xrcc5* p3 | TGACTGGCTTTTAGCGGCTC | CGGAAACCTAGAGTGTCGCA |
| *Mad2l2* p1 | AGCGTCTCGGTTGCTTCTTA | CAACGTCCCCGGACTCTTAG |
| *Mad2l2* p2 | ACACAGCGATCATACGCAGA | CAAGGAACGAGCTAAACCGC |
| *Mad2l2* p3 | TTCTGGAGTAGCGCGTTAGG | CTCTACGCTCCTCCCCGATT |
|  |  |  |

**Reference**

1	Jiang, S., Knapstein, P., Donat, A., Tsitsilonis, S. & Keller, J. An optimized protocol for a standardized, femoral osteotomy model to study fracture healing in mice. *STAR Protocols* **2**, 100798, doi:<https://doi.org/10.1016/j.xpro.2021.100798> (2021).

2	Saul, D. *et al.* Modulation of fracture healing by the transient accumulation of senescent cells. *eLife* **10**, doi:10.7554/eLife.69958 (2021).

3	Kawamoto, T. & Kawamoto, K. in *Skeletal Development and Repair: Methods and Protocols*	(ed Matthew J. Hilton) 149-164 (Humana Press, 2014).

4	Marecic, O. *et al.* Identification and characterization of an injury-induced skeletal progenitor. *Proceedings of the National Academy of Sciences of the United States of America* **112**, 9920-9925, doi:10.1073/pnas.1513066112 (2015).

5	Rőszer, T., Józsa, T., Kiss-Tóth, É. D., De Clerck, N. & Balogh, L. Leptin receptor deficient diabetic (db/db) mice are compromised in postnatal bone regeneration. *Cell and Tissue Research* **356**, 195-206, doi:10.1007/s00441-013-1768-6 (2014).

6	Ambrosi, T. H. *et al.* Aged skeletal stem cells generate an inflammatory degenerative niche. *Nature* **597**, 256-262, doi:10.1038/s41586-021-03795-7 (2021).

7	Chan, C. K. *et al.* Identification and specification of the mouse skeletal stem cell. *Cell* **160**, 285-298, doi:10.1016/j.cell.2014.12.002 (2015).

8	Gulati, G. S. *et al.* Isolation and functional assessment of mouse skeletal stem cell lineage. *Nature protocols* **13**, 1294-1309, doi:10.1038/nprot.2018.041 (2018).

9	Hewitt, G. *et al.* Telomeres are favoured targets of a persistent DNA damage response in ageing and stress-induced senescence. *Nature communications* **3**, 708, doi:10.1038/ncomms1708 (2012).

10	Anderson, R. *et al.* Length-independent telomere damage drives post-mitotic cardiomyocyte senescence. *The EMBO journal* **38**, doi:10.15252/embj.2018100492 (2019).

11	Xu, S. *et al.* Using clusterProfiler to characterize multiomics data. *Nature protocols* **19**, 3292-3320, doi:10.1038/s41596-024-01020-z (2024).

12	Ramírez, F. *et al.* deepTools2: a next generation web server for deep-sequencing data analysis. *Nucleic acids research* **44**, W160-W165, doi:10.1093/nar/gkw257 (2016).

13	Zhang, Y. *et al.* Model-based analysis of ChIP-Seq (MACS). *Genome biology* **9**, R137, doi:10.1186/gb-2008-9-9-r137 (2008).

14	Lawrence, M. *et al.* Software for Computing and Annotating Genomic Ranges. *PLOS Computational Biology* **9**, e1003118, doi:10.1371/journal.pcbi.1003118 (2013).

15	Wickham, H. *Ggplot2: Elegant graphics for data analysis*. (Springer, 2016).
